# Towards spruce-type photosystem II: consequences of the loss of light-harvesting proteins LHCB3 and LHCB6 in Arabidopsis

**DOI:** 10.1093/plphys/kiab396

**Published:** 2021-09-01

**Authors:** Iva Ilíková, Petr Ilík, Monika Opatíková, Rameez Arshad, Lukáš Nosek, Václav Karlický, Zuzana Kučerová, Pavel Roudnický, Pavel Pospíšil, Dušan Lazár, Jan Bartoš, Roman Kouřil

**Affiliations:** 1 Institute of Experimental Botany of the Czech Academy of Sciences, Centre of the Region Haná for Biotechnological and Agricultural Research, 783 71 Olomouc, Czech Republic; 2 Department of Biophysics, Centre of the Region Haná for Biotechnological and Agricultural Research, Palacký University, 783 71 Olomouc, Czech Republic; 3 Electron Microscopy Group, Groningen Biomolecular Sciences and Biotechnology Institute, University of Groningen, Nijenborgh 7, 9747 AG Groningen, The Netherlands; 4 Department of Physics, Faculty of Science, University of Ostrava, 710 00 Ostrava, Czech Republic; 5 Global Change Research Institute of the Czech Academy of Sciences, 603 00 Brno, Czech Republic; 6 Central European Institute of Technology, Masaryk University, 625 00 Brno, Czech Republic

## Abstract

The largest stable photosystem II (PSII) supercomplex in land plants (C_2_S_2_M_2_) consists of a core complex dimer (C_2_), two strongly (S_2_) and two moderately (M_2_) bound light-harvesting protein (LHCB) trimers attached to C_2_ via monomeric antenna proteins LHCB4–6. Recently, we have shown that LHCB3 and LHCB6, presumably essential for land plants, are missing in Norway spruce (*Picea abies*), which results in a unique structure of its C_2_S_2_M_2_ supercomplex. Here, we performed structure–function characterization of PSII supercomplexes in Arabidopsis (*Arabidopsis thaliana*) mutants *lhcb3*, *lhcb6*, and *lhcb3 lhcb6* to examine the possibility of the formation of the “spruce-type” PSII supercomplex in angiosperms. Unlike in spruce, in Arabidopsis both LHCB3 and LHCB6 are necessary for stable binding of the M trimer to PSII core. The “spruce-type” PSII supercomplex was observed with low abundance only in the *lhcb3* plants and its formation did not require the presence of LHCB4.3, the only LHCB4-type protein in spruce. Electron microscopy analysis of grana membranes revealed that the majority of PSII in *lhcb6* and namely in *lhcb3 lhcb6* mutants were arranged into C_2_S_2_ semi-crystalline arrays, some of which appeared to structurally restrict plastoquinone diffusion. Mutants without LHCB6 were characterized by fast induction of non-photochemical quenching and, on the contrary to the previous *lhcb6* study, by only transient slowdown of electron transport between PSII and PSI. We hypothesize that these functional changes, associated with the arrangement of PSII into C_2_S_2_ arrays in thylakoids, may be important for the photoprotection of both PSI and PSII upon abrupt high-light exposure.

## Teaser

Photosystem II supercomplexes in Arabidopsis lacking antenna proteins LHCB3 and LHCB6 differ from their spruce counterparts and form potentially photoprotective semi-crystalline arrays in thylakoids.

## Introduction

Photosynthesis is a very complex process that relies on a synergistic function of large multi-subunit pigment–protein complexes of photosystem II (PSII) and photosystem I (PSI), which are embedded in specific regions of the thylakoid membrane called grana and stroma lamellae, respectively. Photosystems mediate a light-driven electron transport from water molecules across the thylakoid membrane, leading to the reduction of NADP^+^ to NADPH and generation of a proton gradient across the membrane, subsequently utilized by ATP-synthase in the production of ATP. The basic concepts of photosynthesis are shared by the majority of photosynthesizing organisms and the individual photosynthetic proteins and their organization into higher complexes are usually highly conserved.

In land plants, PSII is present in the form of supercomplexes, consisting of a dimeric core complex (C_2_) and light-harvesting antenna (LHC) II. The light-harvesting system is formed by a variable amount of antenna proteins organized into LHCII trimers (LHCB1–3), which are functionally attached to the core via minor antennae (LHCB4–6). The most abundant light-harvesting protein in land plants is LHCB1, its content being about two-fold and eight-fold higher compared with the other two trimer-forming proteins, LHCB2 and LHCB3 (Peter and Thornber, 1991). LHCB1 and LHCB2 can form both homo- and heterotrimers, while LHCB3 is present only in heterotrimers together with two copies of LHCB1/LHCB2 (Caffarri et al., 2004; Standfuss and Kühlbrandt, 2004). The monomeric LHCB4–6 proteins, which represent a minor fraction of LHCII, mediate a specific association of LHCII trimers to the PSII core complex and are crucial for the formation of the PSII supercomplex. The LHCII trimers are designated as “S” and “M” based on the strength of their binding to the core dimer (strongly and moderately bound, Dekker and Boekema, 2005; Kouřil et al., 2018). The S trimers bind to the core complex with the help of LHCB5 and LHCB4, whereas the binding of the M trimers is mediated by LHCB4 and LHCB6. Apart from the different binding sites, the trimers also differ in their protein composition, as the M trimer specifically contains one copy of LHCB3 monomer (Caffarri et al., 2009; Su et al., 2017; van Bezouwen et al., 2017). The largest stable form of PSII in land plants is the C_2_S_2_M_2_ supercomplex, where C_2_ binds two copies of both the S and M trimers (Dekker and Boekema, 2005; Kouřil et al., 2018). Occasionally, the binding of the “L” (loosely bound) and “N” (naked) trimers can further extend the size of the light-harvesting antenna (Boekema et al., 1999; Kouřil et al., 2020). Our knowledge about the composition and architecture of the C_2_S_2_M_2_ supercomplex has gradually improved and we have gained substantial information about the structural details of interactions between individual subunits and pigment arrangements within the supercomplex monomer (Caffarri et al., 2009; Su et al., 2017; van Bezouwen et al., 2017).

A generally accepted dogma that the composition and structure of PSII supercomplexes is uniform and strongly conserved in all land plants was refuted by our finding that LHCB3 and LHCB6 proteins, which had been considered as essential components of LHCII in land plants, are missing in gymnosperm families Pinaceae and Gnetales (Kouřil et al., 2016). In these plants, in the absence of LHCB3 and LHCB6, the M trimer binds to the C_2_ in a different orientation, which results in a specific form of PSII supercomplex that is unique among land plants (henceforth, termed “spruce-type” in this work; Kouřil et al., 2016). It is currently difficult to speculate what was the evolutionary factor that led to the loss of LHCB3 and LHCB6 in these plant families, as we do not have enough information about the physiological consequences of the absence of these two important proteins. Norway spruce (*Picea abies*) and other representatives of Pinaceae and Gnetales are not very convenient model plants and therefore their photosynthetic performance and characteristics have not been extensively analyzed yet. At the same time, even if such study had been performed, it would be extremely difficult to decipher which features of the photosynthetic response of these plants are linked to the loss of LHCB3 and LHCB6 (i.e. linked to the unique structure of their PSII supercomplex) and which are related to other specific properties of these plant groups, including the loss of LHCB4.1/4.2 (Grebe et al., 2019), the loss of the NDH complex (Nystedt et al., 2013), and the presence of flavodiiron protein (Allahverdiyeva et al., 2015; Ilík et al., 2017). To investigate the putative physiological benefits and drawbacks of the unique composition of the light-harvesting system in Norway spruce, we have attempted to create a first approximation of the “spruce-type” PSII supercomplex in Arabidopsis (*Arabidopsis thaliana*) by preparing a double mutant *lhcb3 lhcb6* line.

Arabidopsis single mutant lines lacking either LHCB6 or LHCB3 have already been characterized and the studies have revealed some interesting properties of these mutants. Analysis of the Arabidopsis *lhcb3* mutant has shown that the absence of LHCB3 is compensated by LHCB1 and/or LHCB2 proteins and that the C_2_S_2_M_2_ supercomplexes can be formed in this mutant, as electron microscopy (EM) of *lhcb3* grana membrane fragments revealed semi-crystalline arrays of C_2_S_2_M_2_ supercomplexes (Damkjær et al., 2009). Even though the resolution of the PSII supercomplex structure in this study was very low, the analysis of these arrays suggested that the position of the M trimer in C_2_S_2_M_2_ is modified, but that its binding to C_2_ is probably still mediated by LHCB6 (Damkjær et al., 2009).

The loss of LHCB6 appears to have a much stronger detrimental effect on the photosynthetic performance of Arabidopsis plants than the loss of LHCB3, as strong reduction of plant growth, permanent limitation of electron transport, and impairment of non-photochemical quenching (NPQ) has been reported in *lhcb6* (Kovács et al., 2006; de Bianchi et al., 2008). The analysis of the *lhcb6* mutant revealed that in Arabidopsis, LHCB6 might be important for the binding of the M trimer to C_2_, as no C_2_S_2_M_2_ supercomplexes were observed in the *lhcb6* mutant (Kovács et al., 2006; Caffarri et al., 2009).

It has been suggested that the strong impairment of photosynthesis in *lhcb6* is not primarily caused by the loss of LHCB6 per se, but that it results from the relatively high proportion of PSII arranged into so-called PSII semi-crystalline arrays, which in turn may result in severe and permanent limitation of plastoquinone (PQ) diffusion between PSII and PSI (de Bianchi et al., 2008). The ability of PSII complexes to form semi-crystalline arrays has been known for a long time, the early evidence coming from freeze-fracture experiments. In one of the first studies, Park and Biggins (1964) have reported that the “quantasomes” (i.e. PSII supercomplexes) could exist in thylakoids in a variety of arrangements, from random through linear arrays to crystalline arrays, although the crystals were reported to be rather rare. Despite the lack of any structural details and limited knowledge on the structure and composition of PSII particles at that time, there have already been data suggesting that there are various types of semi-crystals (Miller et al., 1976; Simpson, 1979; Tsvetkova et al., 1995; Semenova, 1995). Currently, we know that all types of PSII supercomplexes observed in land plants (C_2_S_2_M_2_, C_2_S_2_M, and C_2_S_2_) are able to form semi-crystalline arrays (Boekema et al., 2000; Yakushevska et al., 2001), but the mechanism and regulation of their formation, as well as their physiological function, importance, and putative benefits, are still very poorly understood.

In our study, we have prepared Arabidopsis double mutant line lacking LHCB3 and LHCB6 in an attempt to reproduce the unique “spruce-type” PSII supercomplex in Arabidopsis, which would help us to obtain valuable information about the possible physiological benefits of this type of supercomplex. From the published studies, we already know that in the absence of LHCB6, the “regular” M trimer containing LHCB3 is not able to bind to the supercomplex. Our primary question thus was whether the additional loss of LHCB3 in *lhcb6* mutant line can facilitate the binding of the M trimer to C_2_S_2_ and if not, what could be the possible factors preventing its appearance in Arabidopsis. It appears that indeed, in Arabidopsis the loss of both LHCB3 and LHCB6 is not sufficient for the stable formation of “spruce-type” supercomplex. At the same time, we have found that in the Arabidopsis double mutant *lhcb3 lhcb6*, the majority of PSII are arranged into C_2_S_2_ semi-crystalline arrays. Therefore, we have used this mutant for an extensive analysis of its primary photosynthetic reactions in order to shed some light on the possible physiological/regulatory role of PSII ordering into C_2_S_2_ semi-crystalline arrays.

## Results

### The additional loss of LHCB3 does not change the phenotype of Arabidopsis *lhcb6* mutant

**Figure 1:**
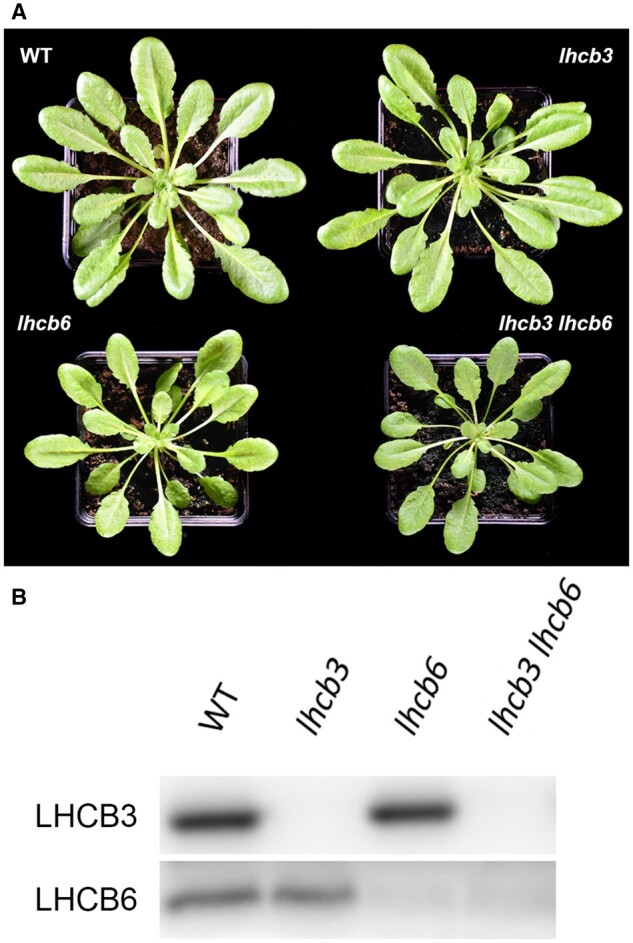
Phenotype and immunoblot analysis. A, Phenotype of *A.* *thaliana* WT and mutant plants (*lhcb3*, *lhcb6*, *lhcb3 lhcb6*) grown in control conditions for 6 weeks (120 µmol photons m^−2^ s^−1^, 22 °C, 8/16 h day/night, and 60% humidity). B, Immunoblot analysis of thylakoid membranes of WT and mutant plants (*lhcb3*, *lhcb6*, *lhcb3 lhcb6*) with antibodies directed against minor light-harvesting proteins LHCB3 and LHCB6.

**Figure 2:**
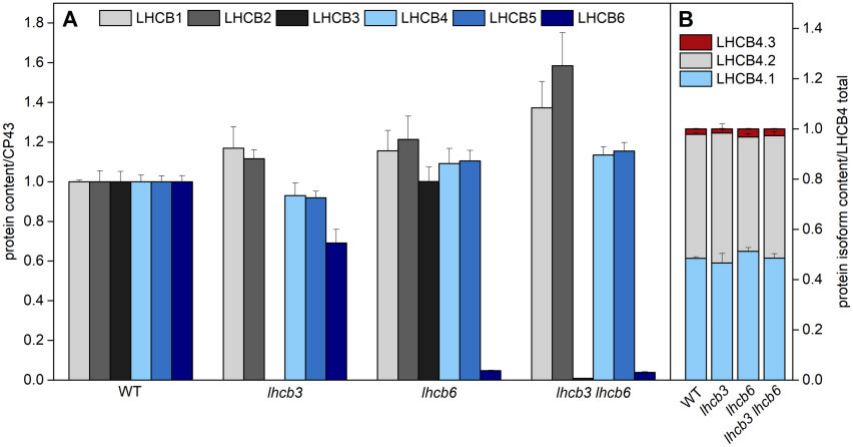
Relative content of light-harvesting proteins in thylakoid membranes of WT and mutant plants (*lhcb3*, *lhcb6*, *lhcb3 lhcb6*). A, The content of individual light-harvesting proteins LHCB1-6 evaluated relatively to the content of chlorophyll protein 43 (CP43, the inner antenna of PSII), and subsequently normalized to WT. B, The content of individual LHCB4 isoforms (LHCB4.1, LHCB4.2, LHCB4.3) related to the sum of all LHCB4 isoforms. The protein content was determined in isolated thylakoid membranes by LC–MS/MS. The presented values are means ± sd from four replicates.

Arabidopsis *lhcb3 lhcb6* double mutant was prepared via the crossing of two SALK T-DNA insertion lines, SALK_020314c (*lhcb3*) and SALK_077953 (*lhcb6*), which were already used in several previous studies. Western blot analysis confirmed a complete absence of LHCB3 in both *lhcb3* and *lhcb3 lhcb6* mutants (Figure 1, B), which agrees with the findings of other authors and confirms that the SALK_020314c is indeed a knockout line (Damkjær et al., 2009; Adamiec et al., 2015). However, in the case of LHCB6, we were able to observe a weak antibody signal in the Western blots, suggesting either cross-reactivity of the used antibody or the presence of some residual amount of LHCB6 in both *lhcb6* and *lhcb3 lhcb6* mutants (Figure 1, B). Closer examination of the T-DNA insertion site in the SALK_077953 line reveals that the T-DNA insertion is localized in the 5’-UTR region of the lhcb6 gene (AT1G15820), which frequently leads to knockdowns rather than knockouts (Wang, 2008). Other authors who have previously used this insertion line and performed Western blots either failed to detect this residual amount of LHCB6 (de Bianchi et al., 2008; Chen et al., 2018), or observed it but disregarded it as non-detectable (3% ± 1% of wild-type (WT) level, Kovács et al., 2006), and therefore the insertion line has been widely used as a knockout mutant for LHCB6. Nevertheless, we have confirmed the presence of the residual amount of LHCB6 in the presumed knockout line also by the mass spectrometry analysis, which revealed a low, but unequivocally detectable amount of LHCB6 in both *lhcb6* and *lhcb3 lhcb6* (4%–5% of WT level, Figure 2, A). Thus, unlike *lhcb3* (SALK_020314c), *lhcb6* (SALK_077953) is a strong knockdown line rather than a complete knockout.

In agreement with a previous study (Damkjær et al., 2009), a phenotypic characterization of mutant plants lacking LHCB3 did not show any distinct changes compared with WT, either in growth rate or pigment composition (Figure 1, A and Table 1). Plants of *lhcb6* line were visibly smaller (Figure 1, A), but their chlorophyll and carotenoid content did not significantly differ from WT and *lhcb3* (Table 1). The double mutant *lhcb3 lhcb6* plants grown under controlled conditions in the phytotron were indistinguishable from the *lhcb6* plants (Figure 1, A), indicating that the additional loss of LHCB3 did not have a substantial effect on the plant visual phenotype.

**Table 1:** Growth parameter and pigment content

	Fresh weight (g)	Chl *a *+* b*	Chl *a*/*b*	Car	Vio	Ant	Zea
WT	1.4 ± 0.2	960 ± 190	2.79 ± 0.09	169 ± 26	15 ± 4	2.0 ± 0.5	ND
*lhcb3*	1.4 ± 0.3	910 ± 73	2.84 ± 0.04	165 ± 8	18 ± 2	2.4 ± 0.4	ND
*lhcb6*	0.6 ± 0.2	820 ±85	2.94 ± 0.07	149 ± 14	18 ± 2	3.1 ± 0.2	ND
*lhcb3 lhcb6*	0.7 ± 0.2	800 ± 68	2.96 ± 0.05	148 ± 12	17 ± 2	3.1 ± 2.1	ND

*Notes*: Presented values are means ± sd. Fresh weight of individual rosettes was measured (*n *=* *13–15). Pigment content is expressed in µg g^−1^ fresh weight (*n *=* *4). Chl, chlorophyll; Car, carotenoids; Vio, violaxanthin; Ant, antheraxanthin; Zea, zeaxanthin; and ND, not detectable.

The changes in LHCB protein levels in individual mutant lines were assessed using mass spectrometry and expressed relative to protein levels in WT. The loss of LHCB3 in *lhcb3* led to a slight increase in the amount of LHCB1 and LHCB2 proteins (Figure 2, A), which probably replace LHCB3 in the M trimer. At the same time, the amount of LHCB6 decreased to approximately 70% of WT level (Figure 2, A), which has not been observed on Western blots from *lhcb3* plants in previous studies (Damkjær et al., 2009; Adamiec et al., 2015). In *lhcb6* mutant plants, we have found again a slight increase in the amount of LHCB1 and LHCB2 (Figure 2, A), which is in agreement with previously observed trends (Kovács et al., 2006; de Bianchi et al., 2008). The level of LHCB3 did not change (Figure 2, A), although literature suggests a decrease to 70% (Kovács et al., 2006; Chen et al., 2018) or even 25% (de Bianchi et al., 2008) of the WT level. LHCB4 and LHCB5 did not show any distinct change in abundance (Figure 2, A). Mass spectrometry analysis confirmed the presence of a residual amount of LHCB6 protein in the *lhcb6* line (less than 5% of the WT level, Figure 2, A), which was already observed in immunoblots (Figure 1, B). The protein composition of the double mutant plants *lhcb3 lhcb6* was similar to *lhcb6* plants, except for the absence of LHCB3 and a slightly more pronounced increase in LHCB1 and LHCB2 abundance (Figure 2, A).

Out of all assessed LHCB proteins, LHCB4 and LHCB5 were affected the least. It is of note that these two proteins can be considered as a part of the “functional core” of the PSII antenna system in all organisms from the green lineage (Alboresi et al., 2008), and unlike the rest of the LHCB proteins, their content is not readily affected by environmental conditions (Ballottari et al., 2007). As LHCB4 is known to be present in Arabidopsis in three isoforms (LHCB4.1, LHCB4.2, and LHCB4.3), we used mass spectrometry to analyze the relative contribution of individual isoforms to the total amount of LHCB4. In WT, LHCB4.1 and LHCB4.2 isoforms were present in approximately equimolar amounts, which agrees with the recently published data (McKenzie et al., 2020), and the loss of LHCB3 and/or LHCB6 did not significantly change this ratio (Figure 2, B). The relative contribution of the third isoform, LHCB4.3, was very low in all analyzed plants (Figure 2, B).

### C_2_S_2_ is the main stable form of PSII supercomplex in Arabidopsis *lhcb3 lhcb6* mutant

**Figure 3:**
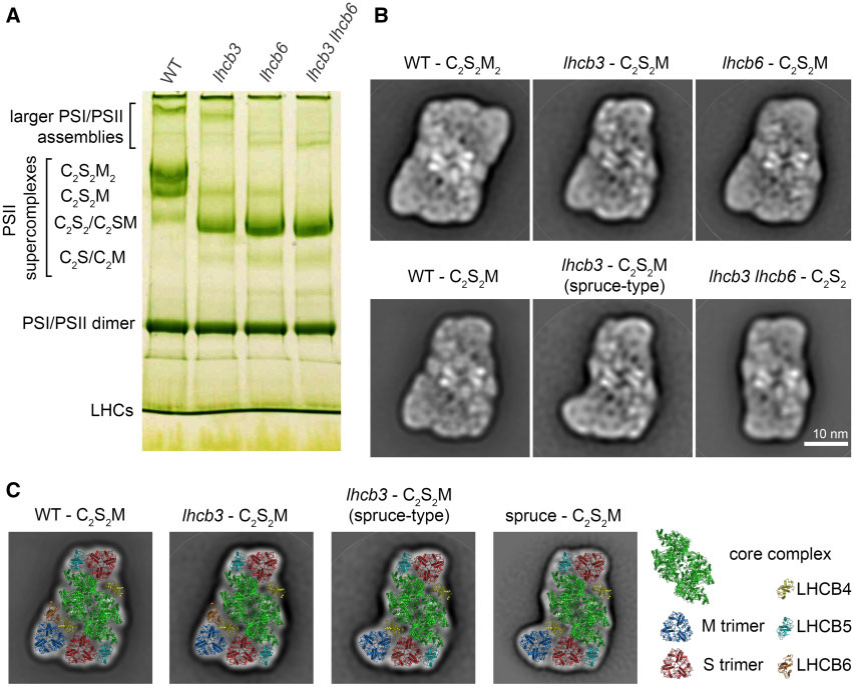
Separation and structural characterization of PSII (PSII) supercomplexes from WT and mutant plants (*lhcb3*, *lhcb6*, *lhcb3 lhcb6*). A, CN polyacrylamide gel electrophoresis separation of pigment–protein complexes from thylakoid membranes from *A.* *thaliana* WT, *lhcb3*, *lhcb6*, and *lhcb3 lhcb6* mutants solubilized by *n*-dodecyl α-d-maltoside. Different forms of separated PSII supercomplexes consist of PSII core dimer (C_2_) and one and/or two copies of strongly (S) and moderately (M) bound light-harvesting trimers. B, Structural characterization of the largest forms of PSII supercomplexes revealed in WT and *lhcb3*, *lhcb6*, and *lhcb3 lhcb6* mutants. In WT, the gel bands designated as C_2_S_2_M_2_ and C_2_S_2_M were analyzed. In *lhcb3* and *lhcb6*, the C_2_S_2_M gel bands, and the C_2_S_2_ gel band in *lhcb3 lhcb6* were analyzed. C, Structural models of C_2_S_2_M PSII supercomplexes from WT and *lhcb3* mutant shown in (B) supplemented with the model of C_2_S_2_M separated from thylakoid membranes of spruce (Kouřil et al. 2016). The models were obtained by a fit of the high-resolution structure from van Bezouwen et al. (2017). Individual PSII subunits are color-coded.

To analyze the impact of the loss of LHCB3 and/or LHCB6 proteins on the formation and structure of PSII supercomplexes in Arabidopsis, we have used clear-native PAGE (CN-PAGE), which enabled us to separate individual photosynthetic protein complexes from thylakoid membranes mildly solubilized with *n*-dodecyl α-d-maltoside (α-DDM). The separation profile of PSII supercomplexes from WT (Figure 3, A) agrees with our previously published data (Nosek et al., 2017) and confirms that C_2_S_2_M_2_ is the most abundant form of PSII supercomplex present in Arabidopsis plants grown under normal light conditions (Kouřil et al., 2013). Other forms (C_2_S_2_M and namely C_2_S_2_/C_2_SM) are not so frequent and may also originate from the disassembly of the C_2_S_2_M_2_ complex during sample preparation.

In plants lacking LHCB3, we did not observe any distinct band that would correspond to C_2_S_2_M_2_, instead, the dominant form appeared to be the small C_2_S_2_ supercomplex (Figure 3, A). However, the presence of a faint, but clearly visible band at the position corresponding to C_2_S_2_M supercomplexes suggests that even in the absence of LHCB3, the M trimer is able to bind to C_2_S_2_. The data obtained by Damkjær et al. (2009) on fragments of granal membranes with crystalline arrays indicate that the C_2_S_2_M_2_ supercomplex is indeed present in *lhcb3* in vivo. It seems that in the absence of LHCB3, the binding of the M trimer to C_2_S_2_ is very weak and the fragile C_2_S_2_M_2_ supercomplexes are easily disrupted to smaller supercomplexes during the solubilization. In the previous report, where sucrose gradient fractionation was used instead of CN-PAGE to analyze *lhcb3* PSII supercomplexes, even the C_2_S_2_M supercomplexes were not detectable and C_2_S_2_/C_2_SM was the only detected form of PSII supercomplex (Caffarri et al., 2009).

The separation profile of PSII supercomplexes isolated from *lhcb6* is very similar to *lhcb3* (Figure 3, A). In agreement with a previous study (Caffarri et al., 2009), we have found out that the band corresponding to C_2_S_2_M_2_ is absent and that the major form of PSII supercomplex in this mutant is C_2_S_2_. However, in addition to this, we were able to observe also a faint band at the position of C_2_S_2_M supercomplexes. This again suggests the superiority of our CN-PAGE purification approach, as this form of PSII supercomplexes was not detectable in *lhcb6* via sucrose gradient fractionation (Caffarri et al., 2009). We have assumed that the formation of a small amount of C_2_S_2_M was enabled by the presence of the residual amount of LHCB6 (Figures 1, B, 2, A) in the *lhcb6* mutant. This hypothesis has been indeed confirmed by mass spectrometry analysis of this band, as it contained LHCB6 and had the same protein composition as C_2_S_2_M from WT plants (Supplemental Figure S1). In contrast to *lhcb3*, in *lhcb6*, the C_2_S_2_M_2_ supercomplexes are probably not present even in the membrane, as the previous EM analysis of PSII supercomplexes in *lhcb6* thylakoids revealed more than 95% of C_2_S_2_ (Kovács et al., 2006). Thus, in the absence of LHCB6, the only stable form of PSII supercomplex appears to be C_2_S_2_ (Figure 3, A).

CN-PAGE analysis of PSII supercomplexes from the double mutant *lhcb3 lhcb6* revealed only one strong PSII band, corresponding to C_2_S_2_ (Figure 3, A). This is interesting in the light of the data obtained from Norway spruce. This representative of Pinaceae lacks both LHCB3 and LHCB6 (Kouřil et al., 2016), but at the same time, the CN-PAGE separation of its thylakoid membranes provides clear evidence of the presence of large forms of PSII supercomplexes (Kouřil et al., 2016, 2020). However, based solely on electrophoretic analysis, it was not possible to decide whether the larger forms of PSII supercomplexes are absent in *lhcb3 lhcb6* mutant or whether they are just too unstable to be isolated via CN-PAGE as in the case of *lhcb3*.

### The appearance of “spruce-type” PSII supercomplex in Arabidopsis *lhcb3* mutant

The largest forms of PSII supercomplexes separated by CN-PAGE from thylakoid membranes of individual lines were analyzed using single-particle EM. The analysis of the supercomplexes from C_2_S_2_M_2_ and C_2_S_2_M WT bands showed the presence of typical forms of supercomplexes (Figure 3, B; Caffarri et al., 2009), which was also confirmed by the fitting of our projection maps with a structural model of PSII supercomplex from Arabidopsis (van Bezouwen et al., 2017) and by detailed protein analysis of individual supercomplexes (Supplemental Figure S1).

The analysis of a faint CN-PAGE band from *lhcb3* that is present at the tentative position of C_2_S_2_M confirmed that it indeed contained C_2_S_2_M supercomplexes. The proteomic data suggest that the absence of LHCB3, normally present in the LHCII trimer at M position, is in *lhcb3* compensated by increased amounts of LHCB1/LHCB2 (Figure 2, A). Detailed single particle EM image analysis revealed the presence of two different forms of the C_2_S_2_M supercomplex in *lhcb3*. About 90% of the particles were represented by a C_2_S_2_M supercomplex where the M trimer binds to the PSII core complex with the help of both LHCB4 and LHCB6 subunits (Figure 3, B and C). This type of supercomplex is similar to the C_2_S_2_M present in WT, the only difference being a slight change in the position of the M trimer with respect to the S trimer (rotation by ca 10°, Figure 3, B and C). This result is in agreement with the previous report, where the change in the orientation of the M trimer was suggested from the analysis of semi-crystalline arrays of C_2_S_2_M_2_ supercomplexes in *lhcb3* granal membranes (Damkjær et al., 2009). In the second type of C_2_S_2_M present in *lhcb3*, however, the M trimer is attached to the core complex only via LHCB4, without the participation of LHCB6. Single particle analysis revealed that in this type of supercomplex, the position normally occupied by LHCB6 is empty (Figure 3, B and C). In this case, the structure closely resembles the C_2_S_2_M supercomplex observed previously in Norway spruce (Kouřil et al., 2016); therefore, we term it “spruce-type.” Although this “spruce-type” supercomplex represents only about 10% of all the analyzed supercomplexes from the *lhcb3* C_2_S_2_M band, it is not possible to draw any reliable conclusions about its natural abundance in *lhcb3* thylakoids. PSII supercomplexes appear to be very fragile in the absence of LHCB3, as evidenced by the disruption of C_2_S_2_M_2_ supercomplexes from *lhcb3* by even very mild solubilization (Figure 3, A). Therefore, any differences in the stability of the two forms of C_2_S_2_M during the preparation of the sample for CN-PAGE and EM can easily distort the estimation of their relative abundance in intact thylakoids. However, irrespective of its relative occurrence in vivo, our finding demonstrates that even in Arabidopsis, the absence of LHCB3 and LHCB6 can lead to the formation of the “spruce-type” C_2_S_2_M supercomplexes. The presence of this LHCB6-less “spruce-type” C_2_S_2_M supercomplex could contribute to the observed decrease in the relative amount of LHCB6 in *lhcb3* thylakoids (Figure 2, A).

Single particle analysis of the C_2_S_2_M band from *lhcb6* revealed a typical form of the C_2_S_2_M supercomplex observed in WT, without any structural modification (Figure 3, B). The projection map shows a density at the position of LHCB6, which could in theory indicate that there is a replacement of LHCB6 by some other LHC protein. However, the mass spectrometry analysis of supercomplexes eluted from this *lhcb6* CN-PAGE band unequivocally confirmed that these C_2_S_2_M supercomplexes indeed contain LHCB6 (Supplemental Figure S1) and that the appearance of the faint C_2_S_2_M band is a direct consequence of the presence of the residual amount of LHCB6. This band would probably be absent in a complete LHCB6 knockout and the only stable form of PSII supercomplex would be C_2_S_2_.

The analysis of the C_2_S_2_ band from *lhcb3 lhcb6* revealed a typical form of C_2_S_2_ (Figure 3, B). Although we have confirmed the ability of Arabidopsis to form “spruce-type” C_2_S_2_M (see above, Figure 3, B and C), these larger forms of PSII supercomplexes were completely absent in the CN-PAGE of the thylakoids from the double mutant. We have concluded that either C_2_S_2_M_(2)_ supercomplexes are not formed in this mutant at all, or are present in thylakoid membranes, but are too fragile to be isolated by CN-PAGE. To resolve this issue, it was necessary to perform EM analysis of granal thylakoid membranes.

### The majority of PSII supercomplexes in Arabidopsis *lhcb3 lhcb6* mutant are organized into C_2_S_2_ semi-crystalline arrays

EM analysis of isolated grana membranes of individual Arabidopsis mutant lines can bring additional information about the arrangement of PSII supercomplexes in vivo. In WT plants, the arrangement of PSII supercomplexes in thylakoid membranes is mostly random (Supplemental Figure S2, A), but supercomplexes can also specifically interact to form various megacomplexes (e.g. Nosek et al., 2017). Some of these megacomplexes could originate from the disassembly of semi-crystalline arrays of C_2_S_2_M_2_, which are occasionally present in WT thylakoids (Supplemental Figure S2, A; e.g. Kouřil et al., 2013). The arrangement of PSII supercomplexes into semi-crystalline arrays was observed also in grana membranes isolated from *lhcb3* (Supplemental Figure S2, B). Previously, it has been shown that these arrays have slightly higher abundance in *lhcb3* and that they also consist of C_2_S_2_M_2_ (Damkjær et al., 2009).

**Figure 4:**
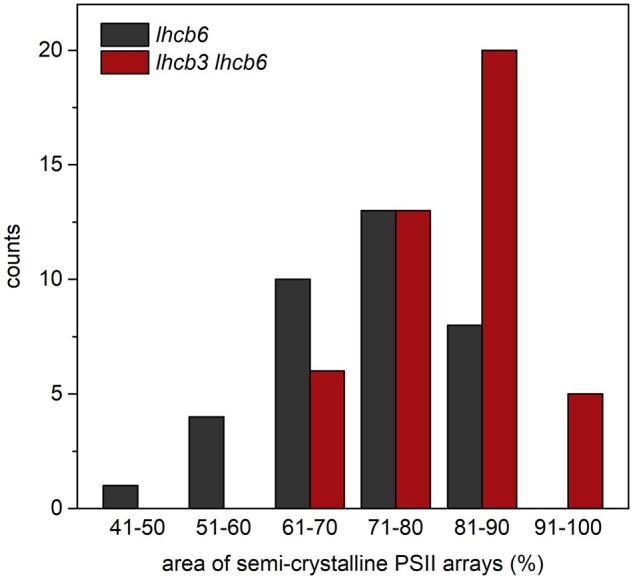
Histogram of a relative representation of two-dimensional semi-crystalline arrays of PSII in the grana membranes from *lhcb6* and *lhcb3 lhcb6* mutants. Area of semi-crystalline arrays of PSII per a total area of the grana membranes was determined in 30 electron micrographs of grana membranes from each type of mutants.

In *lhcb6*, the PSII arrays are formed by C_2_S_2_ rather than C_2_S_2_M_2_ supercomplexes (Kovács et al., 2006; de Bianchi et al., 2008) and their abundance is relatively high (Supplemental Figure S2, C). Based on the analysis of freeze–fracture electron micrographs of *lhcb6* thylakoids, the fraction of PSII present in arrays has been previously estimated to be 25% (Goral et al., 2012). Our EM data, however, suggest that the arrays are much more frequent. In the majority of electron micrographs randomly selected for analysis, semi-crystalline arrays were present in 60%–90% of the area of granal thylakoid membranes of *lhcb6* (Figure 4). The remaining membrane areas without arrays were usually represented by low PSII density regions, where randomly oriented PSII complexes were surrounded by seemingly free space (low PSII density areas, Supplemental Figure S2, C). These parts of the granal membrane most likely contained free LHCII trimers, which could not be directly resolved in the membrane by EM due to their low contrast.

**Figure 5:**
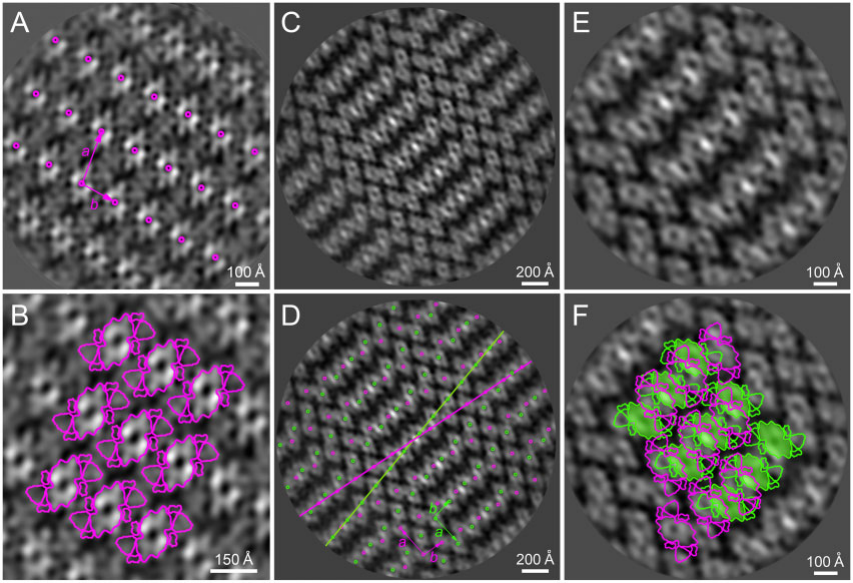
Different types of the two-dimensional semi-crystalline arrays of PSII in the grana membranes from *lhcb3 lhcb6* mutant. A, Image analysis of grana membrane sub-areas (1,320 × 1,320 Å) with semi-crystalline array of PSII complexes revealed a projection map of one layer of PSII complexes ordered into the regular array viewed from the lumen side. The centers of the PSII core complexes represent the lattice points of the PSII arrays (magenta). The unit cell is defined by the *a* and *b* parameters. B, Enlarged central parts of the PSII arrays shown in A. The cartoon model shows that the semi-crystalline array is formed by PSII C_2_S_2_ supercomplexes viewed from the lumen side. C, Image analysis of larger sub-areas (2,160 × 2,160 Å) of another type of semi-crystalline array, a carpet-like motive, of PSII complexes revealed a projection map of two translationally and rotationally shifted layers of ordered PSII complexes. D, The lattice points of the PSII arrays viewed from the lumen and stroma sides are indicated by magenta and green points, respectively. A mutual rotation of the two layers is indicated by the two magenta and green lines and it is about 17°. The unit cells are defined by the *a* and *b* parameters. E, Image analysis focused on smaller sub-areas (1,230 × 1,230 Å) of the semi-crystalline array presented in (C) revealed interactions between the two PSII layers in more details. F, A cartoon model shows variable interactions between PSII C_2_S_2_ supercomplexes from the two adjacent layers viewed from the lumen and stroma sides (in magenta and green, respectively).

**Figure 6:**
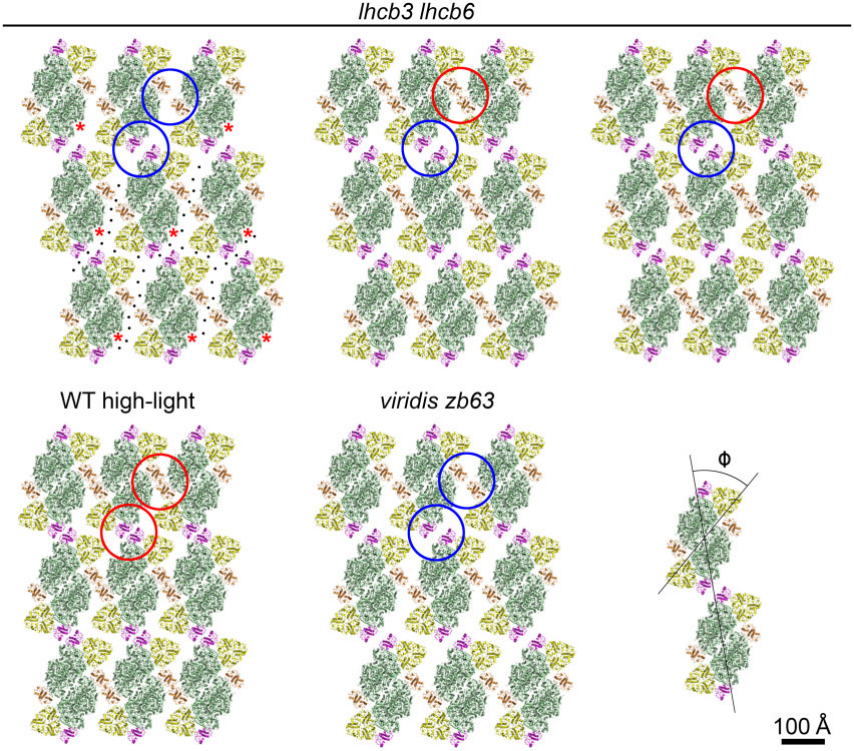
Structural models of different types of packing of PSII C_2_S_2_ complexes into two-dimensional semi-crystalline arrays. A–C, Structural models of C_2_S_2_ arrays in grana membranes of Arabidopsis *lhcb3 lhcb6* mutant with different lattice unit cell parameters (Table 2). The model (A) represents an open conformation with a larger distance between LHCB4 and LHCB5 proteins of neighboring C_2_S_2_ supercomplexes (see blue circles), which is favorable for free diffusion of PQ molecules (black on-scale dots) to/from the *Q*_B_ binding pockets (indicated by red asterisks). On the contrary, the models (B) and (C) show a closer contact between the neighboring C_2_S_2_ supercomplexes, especially between LHCB4 proteins (see red circles), which can hamper a free diffusion of PQ molecules. D, Structural model of C_2_S_2_ arrays in Arabidopsis WT acclimated to high-light intensity indicates even closer contact between neighboring C_2_S_2_ supercomplexes (adopted from Kouřil et al., 2013). E, Structural model of C_2_S_2_ arrays in barley mutant, *viridis zb63*, grown under optimal light conditions shows an open conformation similar to the open conformation (A) in Arabidopsis *lhcb3 lhcb6*. F, Determination of the *ϕ* angle, which is defined as the angle between the vector *a* of the lattice unit cell and the diagonal of the C_2_S_2_ supercomplex. Lattice unit cell parameters of all presented models are shown in Table 2. Structural model of the C_2_S_2_ supercomplex was obtained from Wei et al. (2016). Individual PSII subunits are color-coded: dark green, core complex; yellow, S trimer; magenta, LHCB5; and orange, LHCB4.

In the grana membranes isolated from the double mutant *lhcb3 lhcb6*, the degree of the arrangement of PSII supercomplexes into arrays was very high, with only a minor representation of LHCII-rich regions (Supplemental Figure S3). In most of the analyzed granal membranes, 80%–100% of the area was occupied by semi-crystalline arrays and we did not observe any membrane where the relative contribution of the arrays was lower than 60% (Figure 4). The average parameters of the lattice unit cell of these arrays, calculated from the analyzed crystals, were (243 ± 4) × (165 ± 3) Å with lattice angles 82° or 98° ± 2°. Very similar lattice parameters were reported for C_2_S_2_ arrays in PSI-less *viridis zb63* barley (*Hordeum vulgare*) mutant (250 × 165 Å, angles 80° and 100°; Morosinotto et al., 2006), and also the C_2_S_2_ arrays observed in *lhcb6* appear to be of the same type (de Bianchi et al., 2008). Indeed, fitting of the arrays in *lhcb3 lhcb6* (Figure 5) by a cartoon model of PSII supercomplex (Figure 6) confirms that they consist of C_2_S_2_. Based on this observation, we can conclude that the absence of the C_2_S_2_M_2_ band in CN-PAGE of *lhcb3 lhcb6* (Figure 3, A) cannot be ascribed to the disintegration of the large supercomplexes during sample preparation and that, on the contrary to *lhcb3*, the C_2_S_2_M_(2)_ supercomplexes are not present even in the granal membranes of *lhcb3 lhcb6*.

Previously, it has been shown that in barley *viridis zb63* mutant, PSII can be arranged in several crystal forms (Stoylova et al., 2000), and therefore we have performed a detailed analysis of crystalline arrays in order to find out whether such variability exists also in *lhcb3 lhcb6* mutant. Indeed, we have identified at least three types of C_2_S_2_ arrays, which differ in the dimensions and angle of the lattice unit cell, whereas the tilting of the C_2_S_2_ with respect to the vector of the lattice cell was similar in all three types (Figure 6 and Table 2). The models of individual crystal forms clearly show that although all of them consist of C_2_S_2_, the different tightness of supercomplex packing is likely to have different consequences for the diffusion rate of PQ molecules. Therefore, different types of C_2_S_2_ crystal arrangement can have different effects on PSII photochemical activity.

**Table 2:** Lattice unit cell parameters of C_2_S_2_ arrays in Arabidopsis *lhcb3 lhcb6*, Arabidopsis WT acclimated to high light and barley *vir-zb63* mutant

Plant	*a* (Å)	*b* (Å)	*α* (°)	*ϕ* (°)^a^	Type of C_2_S_2_ double layer	Source
Arabidopsis *lhcb3 lhcb6*	244	170	80	50	Carpet	This study, Figure 6, A
	233	162	84	51	Regular	This study, Figure 6, B
	245	155	83	50	Regular	This study, Figure 6, C
Arabidopsis WT (high light)	234 ± 5^b^	154 ± 1^b^	86 ± 1^b^	48	–	This study, Figure 6, D (data from Kouřil et al. 2013)
Barley *vir-zb63*	250	165	80	*–*	–	Morosinotto et al. (2006)
	244 ± 3^b^	170 ± 3^b^	82 ± 0^b^	51	–	This study, Figure 6, E
Barley *vir-zb63* (far-red light)	234 ± 0.3^b^	162 ± 0.2^b^	81.2 ± 1.9^b^	*–*	*–*	Stoylova et al. (2000)
	243 ± 0.4^b^	162 ± 0.2^b^	81.1 ± 1.9^b^	*–*	*–*	Stoylova et al. (2000)
	235 ± 0.4^b^	158 ± 0.3^b^	80.5 ± 2.0^b^	*–*	*–*	Stoylova et al. (2000)
	215 ± 0.7^b^	175 ± 0.5^b^	87.1 ± 1.7^b^	*–*	*–*	Stoylova et al. (2000)

*Notes*: The *a* and *b* are lengths of vectors of lattice unit cells, *α* is the angle between these vectors, and *ϕ* is the angle between the *a* vector and the diagonal of C_2_S_2_ supercomplex (see Figure 6, F). Data for the evaluation of the lattice unit cell parameters for Arabidopsis acclimated to high light (800 μmol photons m^−2^ s^−1^) was taken from Kouřil et al. (2013). For other details, see the legend to Figure 6 and Materials and Methods.

^a^ Evaluated from the models presented in Figure 6.

^b^ Values ± sd.

The analysis of EM micrographs of granal thylakoid membranes from *lhcb3 lhcb6* (Supplemental Figures S3–S5) also revealed the presence of membrane stacks consisting of pairs of membrane layers interacting through their stromal sides. The layers are attached to each other via two types of interactions between PSII supercomplexes—regular and variable. Regular interactions between PSII supercomplexes in adjacent layers lead to a regular pattern in EM micrographs (Figure 5, A and B and Supplemental Figure S4), which closely resembles the pattern already observed in *viridis zb63* barley mutant (Morosinotto et al., 2006). It remains an open question which component mediates the interaction of PSII supercomplexes over the stromal gap. Unfortunately, in the regular arrays, the mutual orientation of PSII supercomplexes in the interacting layers is difficult to analyze because the interacting supercomplexes vertically overlap each other. It has been suggested that the stacking might be mediated by interactions between adjacent PSII core complexes (PSII sandwiches, Albanese et al., 2016b, 2017) or between overlapping S trimers (Grinzato et al., 2020). Except for the regular arrays, where all PSII supercomplexes in one layer appear to interact in a periodically repeating manner with their counterparts in the second layer, we have also observed “variable” arrays. In these arrays, the interactions between PSII supercomplexes in the adjacent membrane layers are less specific, as the vertically overlapping (i.e. potentially interacting) proteins are variable. These arrays originate via interaction of two translationally and rotationally (about 17°) shifted layers of PSII complexes and are recognizable through the appearance of a “carpet-like” motive in electron micrographs (Figure 5, B–E and Supplemental Figure S5).

### C_2_S_2_ arrays are present in vivo in Arabidopsis *lhcb6* and *lhcb3 lhcb6* mutants

Higher organization of photosynthetic complexes can be sometimes affected by isolation procedures, which are necessary for the preparation of samples for EM. Therefore, we have complemented our study by circular dichroism (CD) spectroscopy, a method that can be used to assess the macroorganization of pigment–protein complexes in thylakoid membrane in vivo (Garab and van Amerongen, 2009). Complex systems such as granal thylakoid membranes provide a complex CD spectrum, consisting of a superposition of signals induced by the intrinsic asymmetry of molecules, excitonic short-range interactions, and so-called psi-type signals (polymer and salt-induced; Garab and van Amerongen, 2009). We are mostly interested in the psi-type signals, as they originate from three-dimensional aggregates, which contain a high number of interacting chromophores and whose dimensions are comparable to the wavelength of measuring light.

**Figure 7:**
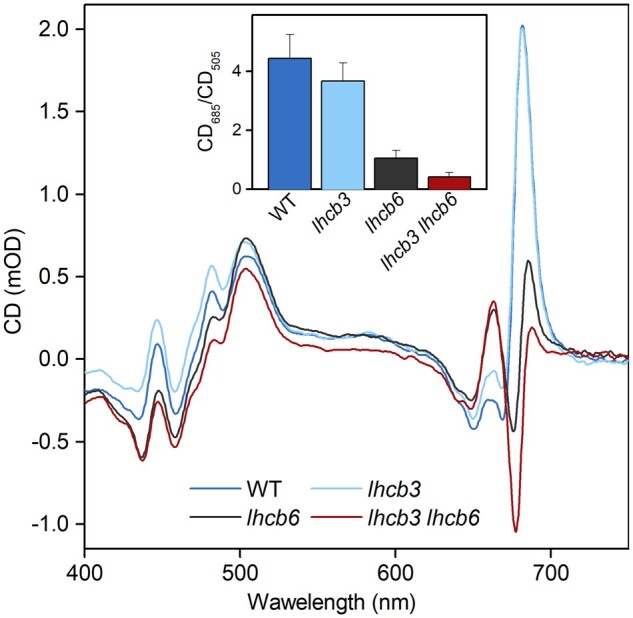
CD Spectra of intact leaves of WT and mutant plants (*lhcb3*, *lhcb6*, *lhcb3 lhcb6*). Averaged spectra from four leaves of each genotype (WT, *lhcb3*, *lhcb6*, *lhcb3 lhcb6*) are shown, for each leaf three scans were collected and averaged. CD spectra were normalized to the Chl *Q*_y_ absorption band. The inset presents the ratio of amplitudes of positive psi-type CD bands (CD_685_/CD_505_), mean ± sd is shown (*n* = 4).

The main spectral features of the CD spectra of WT leaves (Figure 7) are three bands at wavelengths around (+)685, (−)673, and (+)505 nm, which are of psi-type origin and thus reflecting the supramolecular organization of pigment–protein complexes (Barzda et al., 1994; Dobrikova et al., 2003). It is well established that the (+)685 and (−)673 nm psi-type CD bands are associated with chlorophyll molecules while the (+)505 psi-type CD band mainly originates from a β-carotene bound to PSII core complexes (Kovács et al., 2006; Tóth et al., 2016). The psi-type CD (−)673 nm band is mostly associated with grana stacking (Garab et al., 1991), whereas the (+)505 and (+)685 nm bands are not linked directly to the granal stacking, but rather to the lateral supramolecular organization of PSII–LHCII supercomplexes (Kovács et al., 2006; Tóth et al., 2016).

In agreement with EM microscopy analysis, the absence of LHCB3 did not lead to a substantial change in long-range macroorganization of the thylakoid membranes compared with WT, which is evidenced by a very similar psi-type signal (Figure 7). On the other hand, the depletion of LHCB6 (in both *lhcb6* and *lhcb3 lhcb6*) led to an almost complete loss of the main positive psi-type band at 685 nm, whereas the 505-nm band was unaffected (Figure 7). This change, which agrees with previously published data obtained on *lhcb6* (Kovács et al., 2006; Tóth et al., 2016), was even more pronounced in leaves of *lhcb3 lhcb6* (Figure 7). The ratio CD_685_/CD_505_, suggested to be proportional to PSII nearest neighbor distance (Tóth et al., 2016), is also lower in the double mutant compared with *lhcb6* (Figure 7). CD data thus support our finding from EM analysis that the *lhcb3 lhcb6* mutants have an exceptionally high abundance of C_2_S_2_ semi-crystalline arrays in their granal thylakoid membranes.

### C_2_S_2_ arrays transiently slow down the electron flow from PSII to PSI

To examine the functional state of the donor and acceptor sides of the PSII complex in the LHCB mutants, the kinetics of the *Q*_A_^−^ reoxidation after a single-turnover saturating flash was measured on intact leaves (Supplemental Figure S6). For WT and mutant leaves, we have observed very similar multiphasic fluorescence decay kinetics of variable fluorescence, which could be deconvoluted into three different exponential decays (Supplemental Table S1). The fast decay component (time constant ∼460–490 µs, relative amplitude ∼ 62%–65%), which arises from *Q*_A_^−^ to *Q*_B_/*Q*_B_^−^ electron transfer (Vass et al., 1999), was similar in all plants, which indicates that the redox gap between the two quinone acceptors is largely unaffected in the studied LHCB mutants. The middle decay phase (∼70–80 ms, 14%–16%), reflecting the *Q*_A_^−^ reoxidation in the PSII centers with an empty *Q*_B_ pocket (Deák et al., 2014), was also unchanged, suggesting a very similar redox state of PQ in the dark in WT and the mutants. Only very minor differences were observed in the slow phase of the decay (∼3.6–4 s, 20%–23%), arising from S_2_(*Q*_A_*Q*_B_)^−^ charge recombination, indicating that also the donor side of PSII in the mutants is not substantially different from WT. Thus, we can conclude that no substantial changes in the properties of both the acceptor and donor side of PSII were observed.

**Figure 8:**
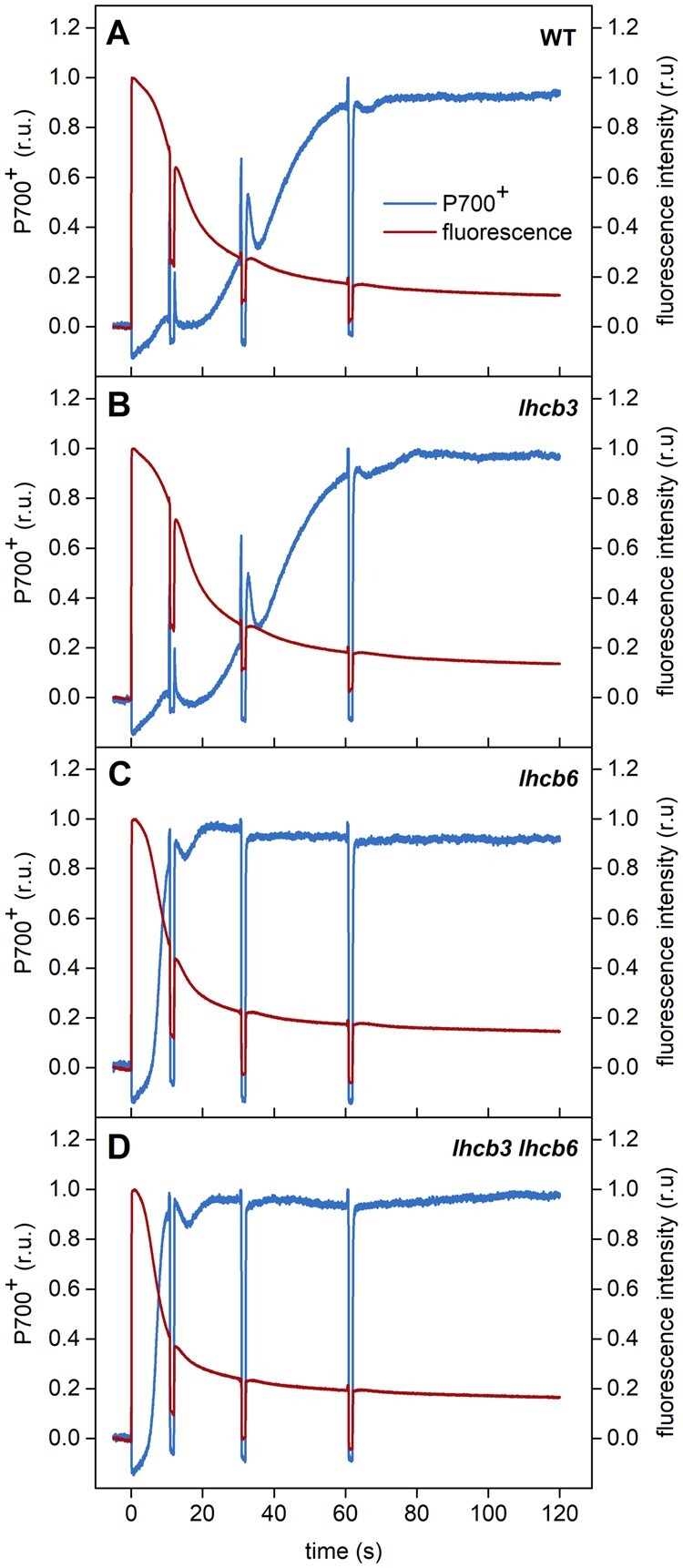
Representative P700 oxidation and chlorophyll fluorescence induction curves of WT and mutant plants (*lhcb3*, *lhcb6*, *lhcb3 lhcb6*). The induction curves were recorded in leaves dark-adapted for 30 min and then exposed to actinic light (800 μmol photons m^−2^ s^−1^). The induction curves were interrupted by saturating red light pulses (300 ms, 10,000 μmol photons m^−2^ s^−1^) followed by switching off the actinic light for 1 s, which were necessary for the calculation of PSII and PSI activity parameters presented in Figure 9. Representative curves are shown.

Although the function of PSII per se was not affected by the loss of LHCB3 and/or LHCB6 or by the arrangement of PSII into C_2_S_2_ arrays, the mesoscopic arrangement of PSII has important consequences for the functionality of the electron transport chain. To analyze the electron transport balance between PSII and PSI, we have simultaneously measured chlorophyll fluorescence and P700 oxidation, reflecting the photochemical activity of PSII and PSI (Figure 8). Upon switching on the actinic light, P700 is gradually oxidized due to PSI photochemistry and subsequent outflow of electrons from the acceptor side of PSI, but at the same time, it is reduced by electrons from PSII or cyclic electron flow around PSI. The balance between these processes shapes the final P700 signal.

**Figure 9:**
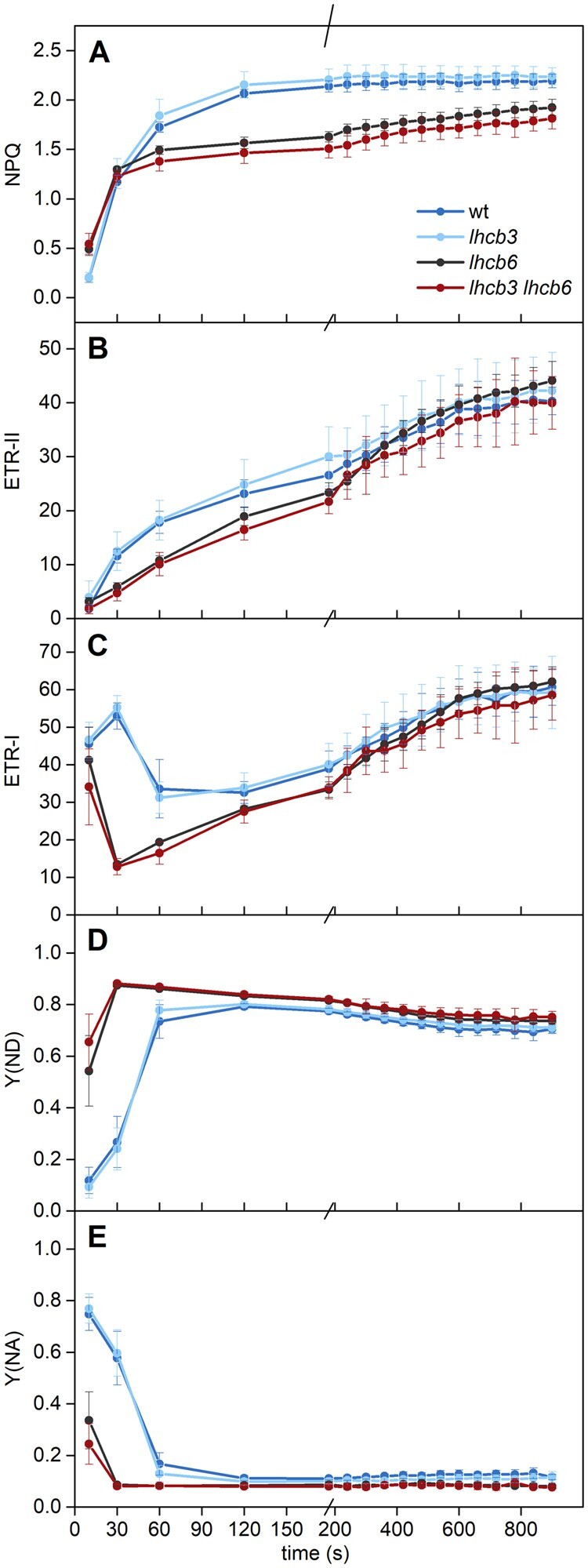
Photosynthetic control parameters in WT and mutant plants (*lhcb3*, *lhcb6*, *lhcb3 lhcb6*) during light exposure. The parameters of (A) NPQ in PSII, electron transport rates through (B) PSII and (C) PSI (ETR-II and ETR-I), and quantum yields of non-photochemical energy dissipation in PSI due to (D) donor and (E) acceptor side limitation (Y(ND) and Y(NA)) were recorded in leaves dark-adapted for 30 min and then exposed to actinic light (800 μmol photons m^−2^ s^−1^). The parameters were calculated using saturating red light pulses (300 ms, 10,000 μmol photons m^−2^ s^−1^) applied during 16-min actinic light exposure. The presented values are means ± sd from four plants.

In WT leaves, the full stable oxidation of P700 is reached after ca 60 s of light exposure (Figure 8, A). Within this period, the limitation of electron flow at the acceptor side of PSI is replaced by the limitation of electron flow at the donor side of PSI (Figure 9, D and E). This response probably reflects the induction of so-called photosynthetic control, that is the slowing of electron flow on the level of the cytochrome b_6_f complex due to lumen acidification induced by cyclic electron flow around PSI (Yamamoto and Shikanai, 2019). The P700 kinetics is the same in the leaves of *lhcb3* mutants (Figure 8, B), indicating that the electron transport is similar to WT plants. In *lhcb6* and *lhcb3 lhcb6* mutants, however, the light-induced oxidation of P700 is much faster, as P700 is fully oxidized already within the first ca 15 s of illumination (Figure 8, C and D). Although in principle this type of response can result from faster activation of electron outflow from the acceptor side of PSI, the most likely explanation of this phenomenon is the limited supply of electrons to PSI. A very similar acceleration of P700 oxidation upon transition to high light was observed also in *pgr1* Arabidopsis mutant (Yamamoto and Shikanai, 2019), in which the sensitivity of the activation of photosynthetic control to lumen acidification is enhanced due to a mutation of the Rieske protein in the cytochrome b_6_f complex (Munekage et al., 2001).

**Figure 10:**
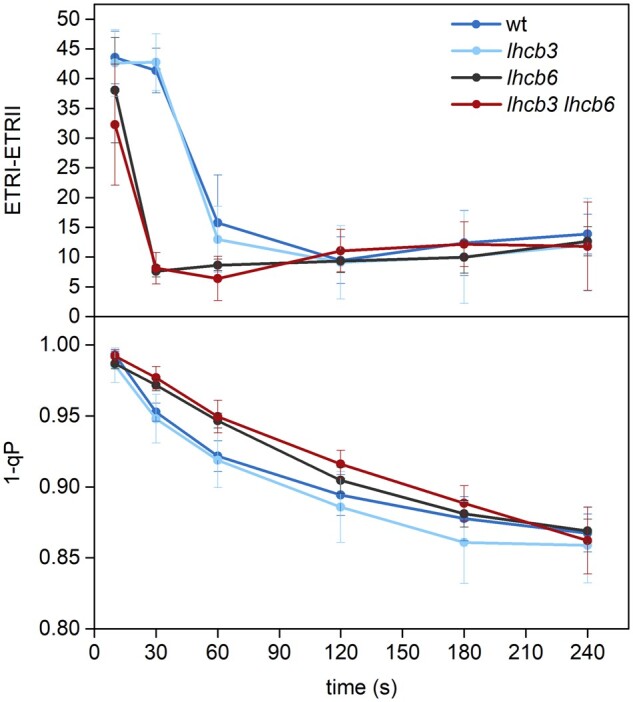
Cyclic electron flow around PSI and redox state of the PQ pool in WT and mutant plants (*lhcb3*, *lhcb6*, *lhcb3 lhcb6*). The extent of cyclic electron flow around PSI was estimated as a difference between ETR-I and ETR-II shown in Figure 9. The fraction of reduced PQ pool in thylakoid membranes was evaluated as 1−qP, where qP is the photochemical quenching coefficient calculated as (*F*_M_′−*F*)/(*F*_M_′−*F*_O_′). The intensity of actinic light was 800 μmol photons m^−2^ s^−1^. The presented values are means ± sd from four plants.

The fast activation of photosynthetic control in mutants without LHCB6 is supported by the highly retarded electron transport rate through PSI (ETR-I, Figure 9, C), accompanied by a strong limitation of electron transport on the donor side of PSI (Y(ND), Figure 9, D). Chlorophyll fluorescence data used for the monitoring of the PSII functioning show that in the first minutes of light exposure, the electron transport rate of PSII is reduced (ETR-II, Figure 9, B). Taking into account that the PSII function per se is not affected by the mutations (see above), these lower values of ETR-II indicate a higher reduction of the PQ pool. In principle, the lower ETR-II could also reflect a lower supply of excitations to PSII due to the smaller light-harvesting capacity of the mutants, but this was not confirmed (see below). The higher values of the parameter 1−*qP* (reflecting higher reduction of PQ pool) in the first minutes of light exposure in *lhcb6* and *lhcb3 lhcb6* (Figure 10) further support the view that in this time range, the PQ pool in thylakoid membranes of *lhcb6* and *lhcb3 lhcb6* is more reduced than in WT and *lhcb3*. The discrepancy between the transient higher reduction of PQ pool and the pronounced limitation of PSI electron transport due to a shortage of electrons on the donor side of PSI in *lhcb6* and *lhcb3 lhcb6* could be a result of the organization of PSII into C_2_S_2_ arrays in these mutants. However, it is important to stress that this restriction is only transient, as the PSII and PSI electron transport rates reach the WT values after several minutes of light exposure (Figure 9, B and C). This result agrees with the results by Chen et al. (2018), who observed WT level of ETR-II at steady-state conditions in *lhcb6* mutant, but disagrees with the results by de Bianchi et al. (2008), who observed in *lhcb6* mutant a permanent restriction of electron transport.

The induction of NPQ of excitations in *lhcb6* and *lhcb3 lhcb6* mutants was different from the WT and *lhcb3*. The absence of LHCB6 resulted in fast induction of NPQ in the first 30 s of light exposure, even faster than in WT and *lhcb3*, followed by a slow rise in NPQ till the end of light exposure (Figure 9, A), at which point its value reached 80%–85% of the WT and *lhcb3* value. Again, these findings correspond with the results by Chen et al. (2018), who observed similar level of the steady-state NPQ in *lhcb6* and WT, but disagrees with the results obtained by de Bianchi et al. (2008), who have reported much lower NPQ values. de Bianchi et al. (2008) explained their NPQ data by permanent restriction in electron transport leading to lower lumen acidification. However, our NPQ data indicate that there is only transient limitation in electron transport rates upon the dark-to-light transition described above.

### The effective antenna size of PSII is not reduced in the mutants

The data from CN-PAGE and EM show that PSII supercomplexes in *lhcb6* and *lhcb3 lhcb6* lack the M trimer, that is the supercomplexes have smaller apparent antenna compared with WT. However, at the same time, our mass spectrometry data clearly indicate that in all mutants, the relative amount of light-harvesting proteins per RC PSII is very similar to WT (Figure 2, A). We can thus ask where these unbound LHCII trimers are located and whether they are functionally connected to PSII. As they cannot be present within the C_2_S_2_ arrays in *lhcb6* and *lhcb3 lhcb6*, they are most probably concentrated in areas with low PSII density sometimes observed at the edge of the arrays (low PSII density area, see Supplemental Figures S2, C, S3). The question is whether they are functionally attached to PSII in crystalline arrays.

To resolve this issue, we have decided to estimate the effective antenna size of PSII via the measurement of chlorophyll fluorescence induction curves. The maximal quantum yield of PSII photochemistry for dark-adapted samples, expressed as the *F*_V_/*F*_M_ ratio (Kitajima and Butler, 1975; Lazár, 1999), is ≈0.83, which is a typical value for healthy leaves (Björkman and Demmig, 1987; Lazár and Nauš, 1998). The mutants without LHCB6 have lower values of the quantum yield, which is caused by a simultaneous increase in minimal chlorophyll fluorescence (*F*_O_) and a decrease in maximal fluorescence (*F*_M_) (Table 3). The effective antenna size, estimated from the O–J phase of the O–J–I–P chlorophyll fluorescence induction curves (TR_0_/RC parameter), was unexpectedly slightly higher in the mutants, and the increase was the highest for the *lhcb3 lhcb6* mutant (119% of WT, Table 3). Similar results have been obtained using estimation of PSII absorption cross-section based on chlorophyll fluorescence induction measured with electron-blocking agent DCMU (ACS PSII). Also, this method indicates that the absorption cross-section of the *lhcb3 lhcb6* mutant is slightly higher than in WT (115% of WT, Table 3).

**Table 3:** Chlorophyll fluorescence induction parameters

	*F*_V_/*F*_M_	*F*_O_ (r.u.)	*F*_M_ (r.u.)	ACS PSII (r.u.)	TR_0_/RC (r.u.)
WT	0.836 ± 0.003	1.00 ± 0.03	1.00 ± 0.03	1.00 ± 0.03	1.00 ± 0.06
*lhcb3*	0.819 ± 0.011	1.07 ± 0.01	0.97 ± 0.05	0.97 ± 0.06	1.04 ± 0.05
*lhcb6*	0.749 ± 0.014	1.33 ± 0.04	0.87 ± 0.06	1.00 ± 0.07	1.06 ± 0.09
*lhcb3 lhcb6*	0.731 ± 0.011	1.41 ± 0.01	0.86 ± 0.05	1.15 ± 0.11	1.19 ± 0.05

*Notes*: Absorption cross-section of PSII was estimated using the area above the chlorophyll fluorescence induction curve in DCMU-treated leaves (ACS PSII) and the initial slope of the O–J rise in the chlorophyll fluorescence induction normalized to the fluorescence intensity at the J step (TR_0_/RC). The parameters, except for the *F*_V_/*F*_M_, are normalized to the values of WT. Presented values are means ± sd (*n *=* *5–8).

Based on our data we can conclude that the domains of unbound LHCII, which we assume to be present at grana margins in *lhcb3 lhcb6*, are most likely responsible for the mild increase in the minimal fluorescence *F*_O_. However, considering the supposed large number of unbound LHCII, they are either very effectively quenched, or are functionally connected to PSII arrays. The estimation of absorption cross-section indicates that the latter possibility is more likely. If only S trimers were involved in light-harvesting, the absorption cross-section should be considerably smaller than in WT, where the predominant form of PSII is C_2_S_2_M_2_. The fact that the absorption cross-section in the mutants without LHCB6 did not decrease compared with WT is a clear indication that the pool of unbound LHCII can supply excitations to PSII in the arrays. The existence of a large fraction of LHCII weakly connected to PSII in thylakoid membranes of the *lhcb6* mutant is also supported by fluorescence lifetime measurements at different excitation wavelengths performed earlier for *lhcb6* mutant by van Oort et al. (2010). These weakly bound LHCIIs characterized by high fluorescence lifetime are probably the reason for the observed higher *F*_O_ values in *lhcb6* and *lhcb3 lhcb6* mutants, which can lower the *F*_V_/*F*_M_ ratio and thus underestimate the real maximum yield of PSII photochemistry.

As the effective antenna of PSII, as well as the functionality of RC PSII, are very similar in WT and all mutants, the transient retardation of the electron transport observed in *lhcb6* and *lhcb3 lhcb6* is most likely a result of limited mobility of electron carriers involved in the transfer of electrons to PSI. In our case, cytochrome b_6_f complex is most likely localized at grana margins (it is probably not a part of arrays) and thus it seems unlikely that the transfer from cytochrome b_6_f complex to PSI via plastocyanin would be largely affected. The most probable electron carrier that would be affected by the arrangement of PSII into semi-crystalline arrays is PQ (Morosinotto et al., 2006; de Bianchi et al., 2008).

### Lateral separation of LHCII results in faster state transitions

It can be expected that the lateral separation of PSII supercomplexes from a substantial part of LHCII trimers would have functional consequences, namely on processes that largely involve LHCII. Assuming that free LHCII trimers are preferentially localized at the periphery of the PSII arrays, likely at grana margins, this localization should affect the process of state transitions. To verify this assumption, we have measured both the extent and rate of state transitions in WT as well as in all the mutants. State transitions were successfully induced in all plants (Supplemental Figure S7) and the changes of absorption cross-sections of PSII upon State I to State II transition were similar in all genotypes (Table 4).

**Table 4:** State transition parameters

	*t*_1/2_ (s)	qT (%)
WT	124 ± 14	11.7 ± 0.4
*lhcb3*	75 ± 12	12.9 ± 0.4
*lhcb6*	26 ± 1	8.7 ± 1.3
*lhcb3 lhcb6*	28 ± 1	9.2 ± 1

*Notes*: The rate of state transition from State I to State II was characterized as the half-time (*t*_1/2_) of a gradual fluorescence decay upon switching off far-red light according to Damkjær et al. (2009). Parameter qT, which reflects the decrease in the LHCII antenna size, was calculated as (*F*_M_′−*F*_M_″)/*F*_M_′. Presented values are means ± sd (*n *=* *5).

However, the rate of state transitions (characterized by *t*_1/2_ of the fluorescence decay upon switching off far-red light) was significantly different in individual mutants. In *lhcb3*, the rate was almost two times faster compared with WT (Table 4). This is in agreement with the previous study, where higher phosphorylation of LHCII (due to the replacement of LHCB3 by LHCB1/2) was identified as a possible cause of faster state transitions (Damkjær et al., 2009). In the *lhcb6* and *lhcb3 lhcb6* mutants, we have found that the rates were about four times faster compared with WT (Table 4), which agrees with faster state transitions in *lhcb6* reported by de Bianchi et al. (2008). As similarly fast state transitions were observed in *lhcb6* and *lhcb3 lhcb6*, it is not likely that the effect is related to the replacement of LHCB3. The reason for such substantially faster state transition in *lhcb6* and *lhcb3 lhcb6* might be the involvement of free LHCII trimers localized at the periphery of the PSII arrays. Due to their peripheral location, they would have a substantially shorter migration distance to PSI than free trimers in WT, where they are probably dispersed in the whole area of the granum. At the same time, the free LHCIIs are most likely co-localized with the cytochrome b_6_f complex, which is required for the activation of the kinase responsible for state transitions. Therefore, once activated, it can directly phosphorylate LHCII located conveniently at its vicinity and the phosphorylated LHCII then can quickly attach to PSI which is located nearby.

## Discussion

### “Spruce-type” C_2_S_2_M supercomplex appears in Arabidopsis lacking LHCB3

Since the emergence of LHCB3 and LHCB6 proteins at the dawn of plant land colonization, the structure of the C_2_S_2_M_2_ supercomplex had been thought to be highly conserved. However, recently we have broken this dogma and have shown that LHCB3 and LHCB6 proteins, whose presence had been considered as a fingerprint of all land plants, are absent in several gymnosperm families. The loss of these two light-harvesting proteins is reflected in a unique structure of PSII supercomplex in these plant species (“spruce-type” C_2_S_2_M_2_ supercomplex). In this supercomplex, the position usually occupied by LHCB6 is empty and the binding of the M trimer to the PSII core complex is modified, resulting in its tighter association with the S trimer (Kouřil et al., 2016).

It is not clear why such specific photosynthetic adaptation has developed in this group of plants. As LHCB3 and LHCB6 proteins are known to be downregulated during high light stress (Kouřil et al., 2013), it is possible that this adaptation could be connected with environmental conditions in which the ancestors of these plant groups have evolved. This would also partially explain the unusual responses of spruce photosynthetic apparatus to changes in light intensity, some of them being typical for high-light adapted plants (Kurasova et al., 2003).

To shed some light on the evolutionary and physiological implications of the “spruce-type” form of PSII supercomplex, we have prepared a double mutant of *A.* *thaliana* lacking LHCB3 and LHCB6 proteins. It has been shown that in the absence of LHCB3, the M trimer can bind to C_2_S_2_, but in a slightly different rotational position than in WT (Damkjær et al., 2009). Nevertheless, this change does not correspond with the binding mode of the M trimer within the “spruce-type” C_2_S_2_M_2_ (Kouřil et al., 2016). At the same time, in Arabidopsis, the C_2_S_2_M_2_ supercomplex with LHCB3-less M trimer is very fragile, a feature not observed in the “spruce-type” supercomplex. The loss of LHCB6 is known to induce much more serious disturbance of photosynthetic apparatus of Arabidopsis than the loss of LHCB3 (Kovács et al., 2006; de Bianchi et al., 2008). It has been shown that the absence of LHCB6 leads to the dissociation of the M trimer, leaving C_2_S_2_ as the main form of supercomplex in the *lhcb6* mutant (Kovács et al., 2006). By creating a double *lhcb3 lhcb6* mutant, we wanted to find out whether the modified LHCII trimer without LHCB3 is able to bind to C_2_S_2_ in the absence of LHCB6 and whether the resulting supercomplex will mimic the structure of the “spruce-type” C_2_S_2_M_2_.

Our results revealed that the “spruce-type” supercomplex without LHCB3 and LHCB6 can be formed even in in Arabidopsis (Figure 3, B), but at the same time, it appears that there are other important factors that are playing role in its formation and stability. Nevertheless, we were able to observe and identify the “spruce-type” supercomplex in an angiosperm plant, in particular in the *lhcb3* mutant. It is obvious that this type of supercomplex can be formed only when LHCB6 is absent along with LHCB3 (Figure 3, B), whereas in the presence of LHCB6, the LHCB3-less supercomplex closely resembles the supercomplex from WT. It is difficult to make any estimation of the abundance of “spruce-type” supercomplex in *lhcb3* in vivo, as the results of CN-PAGE and single particle analysis can be distorted by different stabilities of individual types of supercomplexes. However, the mass spectrometry data show that thylakoid membranes from *lhcb3* have a lower relative amount of LHCB6 (70% of WT, Figure 2, A), so it is tempting to hypothesize that in vivo, the fraction of supercomplexes that lack LHCB6 might correspond to the fraction of “spruce-type” supercomplexes.

We have confirmed that in Arabidopsis, “spruce-type” supercomplexes without LHCB3 and LHCB6 can be formed, but why these supercomplexes are not found in the double mutant *lhcb3 lhcb6*? As has been mentioned, it appears that without LHCB3, the attachment of the M trimer to C_2_S_2_ is much weaker than in WT—in the CN-PAGE of mildly solubilized *lhcb3* thylakoids we did not see any C_2_S_2_M_2_ band (Figure 3, A), although we know that this type of supercomplex is present in vivo in this mutant (Damkjær et al., 2009). No such disassembly was observed for WT C_2_S_2_M_2_ (Figure 3, A). The combined absence of LHCB3 and LHCB6 may lead to so strong destabilization of the M trimer, that the resulting structure is not advantageous. It might be possible that the binding of the M trimer to C_2_S_2_ is so weak that it cannot ensure efficient energy transfer and in such situation, the formation of C_2_S_2_ arrays functionally connected to a large pool of unbound LHCII trimers might represent a preferable, more efficient arrangement.

There is, however, an inevitable question—what is the factor that makes this particular form of supercomplex stable in spruce? It is possible that the key to its different stability in Arabidopsis and spruce is the type of minor antenna protein LHCB4. Due to its prominent position in the PSII supercomplex, LHCB4 is responsible for proper binding of the S and M trimers to the PSII core dimer (de Bianchi et al., 2011; van Bezouwen et al., 2017; Su et al., 2017) and plays a key role in both the energy transfer and PSII photoprotection (de Bianchi et al., 2011). Recently, it has been found that the Pinaceae and Gnetales families lack not only LHCB3 and LHCB6, but also the LHCB4.1/4.2 proteins, which have so far been thought to be the dominant isoforms of LHCB4 in land plants (Grebe et al., 2019). It appears that in spruce, only the gene for the isoform LHCB4.3 (later renamed LHCB8) is present, which has for a long time been considered as a peculiar, rarely expressed gene restricted only to angiosperm clade Eurosids (Klimmek et al., 2006). In spruce PSII, LHCB8 replaces LHCB4.1/4.2 at its binding site in the C_2_S_2_M_2_, which could contribute to the stability of the “spruce-type” supercomplex.

In Arabidopsis, LHCB4 is present in three isoforms coded by three separate genes—LHCB4.1 (AT5G01530), LHCB4.2 (AT3G08940), and LHCB4.3 (AT2G40100). All LHCB4.1/4.2 proteins from various plants have a strictly conserved 15-amino-acid motif at their C-terminus (WxTHLxDPLHTTIxD; Grebe et al., 2019). This motif has been proposed as the site of the interaction of LHCB4.1/4.2 with LHCB6 and LHCB3 (i.e. the M trimer; Su et al., 2017). This C-terminal motif is completely absent in LHCB4.3, which could explain why the evolution pressure in plant species without LHCB3 and LHCB6 led to the loss of LHCB4.1/4.2, whereas LHCB4.3 has been retained. The proteins LHCB4.1 and LHCB4.2 have high sequence homology (89% identity) and similar structure, function, and regulation (Jansson, 1999; de Bianchi et al., 2011), whereas there are strong indications that LHCB4.3 is functionally different from LHCB4.1/4.2, at least in angiosperms (Klimmek et al., 2006).

**Figure 11:**
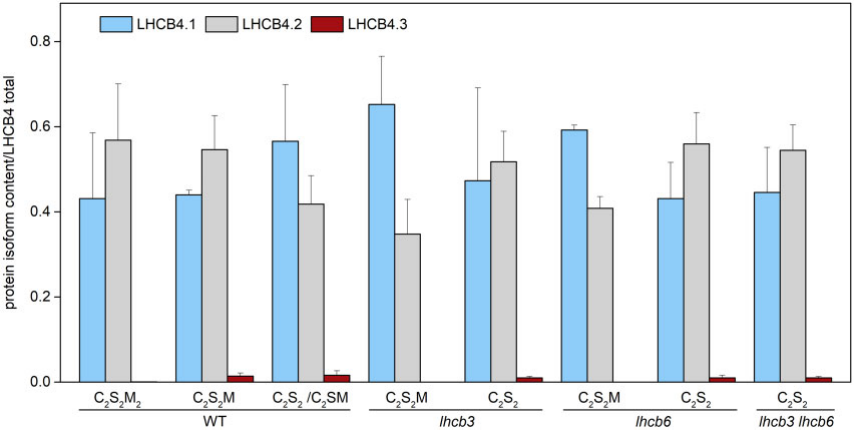
Content of individual LHCB4 isoforms in PSII supercomplexes isolated from *A. thaliana* WT and mutant plants (*lhcb3*, *lhcb6*, *lhcb3 lhcb6*). The relative content of individual LHCB4 isoforms was determined by LC–MS/MS in samples prepared from gel bands excised from CN-PAGE (see Figure 3, A). The contribution of LHCB4.1, 4.2 and 4.3 isoforms, evaluated from normalized PG intensities, is shown relatively to the overall content of LHCB4. The presented values are means ± sd from four replicates. Different forms of separated PSII supercomplexes consist of PSII core dimer (C_2_) and one and/or two copies of strongly (S) and moderately (M) bound light-harvesting trimers.

To explore possible changes in the relative amount of individual LHCB4 isoforms in the mutants, we have performed a detailed analysis of the mass spectrometry data. The amount LHCB4.3 was not increased in any of the mutants compared with WT, which indicates that in Arabidopsis, the loss of LHCB3 and/or LHCB6 does not stimulate the synthesis of LHCB4.3 (Figure 2, B). Even in a very detailed proteomic analysis of the supercomplexes eluted from the *lhcb3* C_2_S_2_M_2_ band, which contains a fraction of “spruce-type” C_2_S_2_M, we did not see any change in the relative LHCB4.3 content (Figure 11). It seems that in Arabidopsis, the regulation of gene expression of *LHCB4.3* is completely different from spruce. The main role of LHCB4.3 in Eurosids is probably related to photoprotection, as there is an up-regulation of the LHCB4.3 gene expression under high-light conditions (Klimmek et al., 2006; Albanese et al., 2016a, 2018, 2019).

We can thus hypothesize that the reason why we were not able to observe “spruce-type” PSII supercomplexes in Arabidopsis *lhcb3 lhcb6* double mutant is the presence of LHCB4.1/4.2 proteins, which are replaced by LHCB4.3 (LHCB8) in spruce. Analysis of the double mutant grown under specific conditions leading to the accumulation of LHCB4.3 might clarify whether LHCB4.3 is indeed the key to the stability of “spruce-type” PSII supercomplex or whether some other factors stabilize this unusual PSII supercomplex structure in spruce (e.g. differences in amino-acid composition of PSII proteins in spruce and Arabidopsis, different phosphorylation pattern, etc.). Physiological characterization of Arabidopsis plants with high abundance of “spruce-type” PSII would be important for our understanding of the specifics of photosynthesis in spruce and could provide valuable clues about the possible evolutionary advantage of the loss of LHCB3 and LHCB6 in some gymnosperm groups.

### Semi-crystalline arrays of PSII supercomplexes

Today we have a lot of information about photosynthetic complexes at the level of individual proteins or protein complexes (high-resolution crystal structures). However, their organization into higher order assemblies and their cooperation within them is still poorly understood, although it is clear that these processes play a key role in the highly organized and strongly regulated process of photosynthesis.

It has been known for a very long time that PSII supercomplexes in granal membranes are able to spontaneously order into semi-crystalline arrays. However, only after solving the PSII supercomplex crystal structure, it was possible to identify particular types of supercomplexes that are involved in array formation. In an extensive study, Boekema et al. (2000) were able to unequivocally distinguish two types of crystals in spinach thylakoids. The more abundant crystals with wider spacing (27.3 × 18.3 nm, 74.5°, unit area 481 nm^2^) were shown to consist of the array of C_2_S_2_M, whereas the rare (1% abundance), more tightly packed crystals (23 × 16.9 nm, unit area of 389 nm^2^) were formed by C_2_S_2_. On the contrary to spinach, the analysis of thylakoid membranes in Arabidopsis revealed only one type of semi-crystalline arrays, which have a bigger unit cell (25.6 × 21.4 nm, 77°, 534 nm^2^) that has been identified as C_2_S_2_M_2_ (Yakushevska et al., 2001). These data indicate that all forms of PSII supercomplexes (i.e. C_2_S_2_, C_2_S_2_M, and C_2_S_2_M_2_) are capable of forming semi-crystalline arrays, although different types of arrays may have different properties and function. Since then, a number of studies have described the presence of the arrays in several plant species grown under various conditions (Kirchhoff et al., 2007; Daum et al., 2010; Sznee et al., 2011; Kouřil et al., 2013; Wientjes et al., 2013; Charuvi et al., 2015) or in various mutants (Ruban et al., 2003; Yakushevska et al., 2003; Morosinotto et al., 2006; Kovács et al., 2006; de Bianchi et al., 2008; Damkjær et al., 2009; Kereïche et al., 2010; Goral et al., 2012; Onoa et al., 2014; Tietz et al., 2015). Thus, it appears that the formation of PSII arrays is a relatively widespread phenomenon.

The heterogeneity in PSII packing, that is the simultaneous existence of “random” and “arrayed” PSII in thylakoid membranes, makes it difficult to analyze the specific properties of PSII in the arrays. The proportion of PSII present in the crystal phase is usually relatively low (around or below 10%), although there are some mutants where the fraction of arrays is reported to be higher (Morosinotto et al., 2006; Goral et al., 2012; Tietz et al., 2015). The double mutant *lhcb3 lhcb6* prepared in this study is unique as it has the majority of PSII supercomplexes packed into C_2_S_2_ semi-crystalline arrays (Figure 4), that is it represents a very good model for the investigation of formation, regulation, and properties of C_2_S_2_ PSII arrays.

It has been suggested that one of the functions of C_2_S_2_ arrays might be related to more efficient energy transfer among different supercomplexes (Morosinotto et al., 2006). As high protein density is a prerequisite for efficient energy transfer (Haferkamp et al., 2010), it can be speculated that the tight packing of C_2_S_2_ supercomplexes improves energy equilibration within interconnected antenna and makes the distribution of excitations to PSII more efficient. As the relative amount of LHCII per reaction center in both *lhcb6* and *lhcb3 lhcb6* is approximately the same as in WT (Figure 2 and Table 1), the LHCII trimers, which in WT are bound within C_2_S_2_M_2_, have to contribute to the pool of “free” LHCII in the mutants. As the free trimers are not part of the arrays, they most likely create LHCII-only areas outside the arrays at the grana margins. The presence of these unattached LHCII is probably responsible for slightly higher minimal fluorescence *F*_O_ observed in the mutants (Table 3). However, it appears that this pool of LHCII has to be functionally connected with PSII in the arrays, as the effective absorption cross-section of PSII in *lhcb6* and *lhcb3 lhcb6* is comparable, or even slightly higher than in WT (Table 3). We can hypothesize that the packing of C_2_S_2_ supercomplexes into arrays and their functional attachment to the pool of free LHCII might represent a compensatory mechanism, which can substitute the binding of M trimers to individual PSII in the form of C_2_S_2_M_2_.

The “separation” of the pool of free LHCII at the grana margins can also explain faster kinetics of state transitions. Due to space restrictions, it is obvious that the cytochrome b_6_f complex is not a part of the arrays and thus it is probably located close to the LHCII pool at grana margins. It has been suggested that the kinase involved in state transitions cannot enter the closely stacked areas in the grana and that it is able to phosphorylate only LHCII in stroma or on the interface between grana and stroma (Dekker and Boekema, 2005). The activation of the kinase thus probably takes place in the same area (or very close) to the pool of free LHCII and, once phosphorylated, LHCII will have a short diffusion distance from the grana margins to stromal thylakoids. As a result, we can expect significantly faster state transition kinetics, which is indeed a phenomenon we have observed in both mutants without LHCB6 (Table 4).

Another of the frequently discussed issues related to PSII arrays is their large effect on the mobility of PQ and thus on the overall electron transport rate. There is an emerging evidence that different types of PSII arrays (C_2_S_2_M_2_ and C_2_S_2_) may actually have the opposite effect on the effectivity of PQ diffusion. It is well known that the granal membrane is highly crowded by proteins (70%–80% of the membrane area, Kirchhoff, 2008) and that the protein concentration is very close to a critical threshold above which the long-range diffusion coefficient drops to zero (Tremmel et al., 2003). It has been proposed that the arrangement of C_2_S_2_M_2_ PSII supercomplexes into crystalline arrays can lead to the formation of a kind of lipidic channel, which might be viewed as a diffusion highway facilitating fast diffusion of PQ molecules to the cytochrome b_6_f complex localized outside the arrays (Tietz et al., 2015). However, on the other hand, it has been argued that in C_2_S_2_ arrays the packing is so tight that there is very limited space for PQ diffusion. Indeed, plants with extensive C_2_S_2_ arrays (Arabidopsis *lhcb6*, barley *viridis zb63*) have been previously reported to suffer from retarded linear electron transport, lower PSII yield, and impaired NPQ (Kovács et al., 2006; Morosinotto et al., 2006; de Bianchi et al., 2008).

However, the situation appears to be much more complex, as a detailed analysis of multiple crystalline arrays in the *lhcb3 lhcb6* mutant revealed that there is a substantial degree of variability in the types of C_2_S_2_ arrays (Table 2). It appears that while some of the C_2_S_2_ arrays are indeed very tightly packed, leaving very small space for the diffusion of PQ, there are also other types of arrays where the diffusion restriction is not so severe. As in all types of the observed crystals the *Q*_B_ pocket (red asterisk, Figure 6) is freely accessible to PQ (black dots, Figure 6), the main factor affecting the PQ diffusion appears to be the spatial “bottleneck” between LHCB4 and LHCB5 in the neighboring supercomplexes (red and blue circles, Figure 6). Figure 6, A shows a type of crystal where the packing of C_2_S_2_ supercomplexes is relatively loose, with enough space left between individual protein complexes to enable diffusion of PQ in all directions. A similar type of crystal was observed in barley mutant without PSI (*viridis zb63*) (Figure 6, E). However, in *lhcb3 lhcb6*, we have also found crystals with smaller lattice units (Table 2), where the PQ diffusion pathway was partially closed due to the very close contact between LHCB4 subunits of neighboring C_2_S_2_ supercomplexes (red circle, Figure 6, B and C). As a result, the PQ diffusion is more restricted in these types of crystals as PQ can move only in one direction (horizontally, considering the crystal orientation shown in Figure 6, B and C). An example of an extremely tightly packed C_2_S_2_ crystal is shown in Figure 6, D. In this PSII arrangement, previously sporadically observed in high-light acclimated WT Arabidopsis (Kouřil et al., 2013), the diffusion of PQ is completely restricted, as the possible pathways are closed in both directions by a tight contact between LHCB4 and LHCB5 antennae (red circles, Figure 6, D). It can be assumed that the PSII supercomplexes arranged into this type of crystal do not contribute to linear electron transport and could represent an operative storage structure of PSII with their own quenching mechanism.

Our data indicate that there is a variety of C_2_S_2_ crystal types, which largely vary with respect to the degree of the restriction of PQ diffusion. It appears that a very small rearrangement of the PSII crystalline arrays can have a large effect on their photochemical activity, as it can lead to the opening or closing of two apparent “bottlenecks” for PQ diffusion, localized between LHCB4 and LHCB5 subunits of the neighboring supercomplexes. It is possible that such crystal rearrangement can be a part of the fine-tuning mechanism by which plants regulate and optimize electron transport under various conditions.

### Formation of C_2_S_2_ arrays—a strategy for the protection of both PSI and PSII upon abrupt high light exposure?

Analysis of the photosynthetic response of *lhcb6* and *lhcb3 lhcb6* mutants revealed significant retardation of the electron transport rate in the first minutes of light exposure. Interestingly, after this transient slow-down, the electron transport parameters in both mutants reached the values of WT and *lhcb3* (Figure 9). This finding contradicts the result obtained by de Bianchi et al. (2008), who have reported that considerable suppression of the PSII electron transport rate of *lhcb6* persists even at steady-state conditions. The reason for the discrepancy is not clear, but it could be associated with different growth conditions leading to substantially higher overall fitness of our *lhcb6* (and *lhcb3 lhcb6*) mutants, which is evidenced by, for example better growth (Figure 1, A), higher *F*_V_/*F*_M_ ratio (Table 3) or NPQ values (Figure 9, A). Obviously, in our case, the transient retardation of electron flow between PSII and PSI in mutants without LHCB6 cannot be attributed to any permanent damage of the electron transport pathway, but seems to be rather a result of altered dynamics of regulatory processes. One of the feasible hypotheses that could possibly explain the dynamic changes in electron transport restriction is the light-induced rearrangement/disordering of the C_2_S_2_ arrays.

It has been shown that immediately after the exposure of dark-adapted plants to light, when the outflow of electrons from the acceptor side of PSI is restricted, the cyclic electron transport (CET) around PSI is highly stimulated (Joliot and Joliot, 2002). As a result, P700 becomes gradually fully oxidized (Figure 8), which is crucial for the protection of PSI against photoinhibition (for a recent review, see Miyake, 2020). The lumen acidification induced by CET then leads to the suppression of electron transport from PSII to PSI on the level of the cytochrome b_6_f complex (photosynthetic control, see Yamamoto and Shikanai, 2019), which, together with the gradual activation of electron outflow at the acceptor side of PSI, leads to the suppression of CET. The activity of CET can be estimated from the difference between electron transport rates ETR I and ETR II and the evaluation of this parameter revealed that the activation and dynamics of CET are the same in WT and *lhcb3* mutant (Figure 10). However, in *lhcb6* and *lhcb3 lhcb6* mutants, the suppression of CET was much faster, within the first 30 s of illumination (Figure 10). The reason for this result is not clear, but could be somehow associated with the transient limitation of PQ diffusion due to the arrangement of PSII into C_2_S_2_ arrays. As the over-reduction of the PQ pool is thought to suppress CET (Allen, 2003; Miyake, 2010), the higher reduction of the PQ pool observed in the first minutes of illumination in *lhcb6* and *lhcb3 lhcb6* (Figure 10) can be connected with the faster inactivation of CET in these mutants. However, it is important to stress that even when CET is activated for a substantially shorter time in *lhcb6* and *lhcb3 lhcb6* mutants, P700 is oxidized in these plants much faster than in WT (Figure 8). It is tentative to speculate that the transient restriction of PQ diffusion, likely resulting from the packing of PSII into C_2_S_2_ arrays, can substitute CET in the photoprotection of PSI.

Another interesting phenomenon that might be connected with the arrangement of PSII in C_2_S_2_ arrays is related to the dynamics of the induction of NPQ. In both *lhcb6* and *lhcb3 lhcb6*, the induction of NPQ in the first seconds of light exposure was faster compared with WT and *lhcb3* (Figure 9, A). The light-induced induction of energy-dependent NPQ (qE) in plants is a strictly regulated and complex process triggered by lumen acidification. Lowering lumen pH leads to several processes, including the dissociation of LHCIIs from PSII supercomplexes, activation of xanthophyll cycle (deepoxidation of violaxanthin (Vio) to zeaxanthin (Zea) by violaxanthin deepoxidase (VDE)), and formation of quenching centers in the aggregated LHCIIs (for a recent review, see Ruban and Wilson, 2020). It has been proposed that the light-induced dissociation of LHCIIs from PSII supercomplexes is represented by a detachment of the M trimer and LHCB6 from the C_2_S_2_M_2_ supercomplex and that this process is induced by protonated PsbS (Betterle et al., 2009; Dall’Osto et al., 2017). As the pK_a_ of PsbS protonation is relatively low (about 5.2, Li et al., 2002), considerably pronounced lumen acidification is necessary for the light-induced disassembly of C_2_S_2_M_2_, that is for the induction of NPQ in plants where C_2_S_2_M_2_ is the major form of PSII supercomplex. As the dissociation of LHCIIs from PSII supercomplexes is necessary to make Vio in LHCIIs available for the conversion to Zea by activated VDE (for a review, see Morosinotto et al., 2003), pronounced lumen acidification (inducing LHCII dissociation) is also required for the effective function of the xanthophyll cycle, although VDE itself is activated already at higher luminal pH (pKa about 6, Günther et al., 1994; Bratt et al., 1995). Therefore, the dynamics of LHCII detachment appears to be the main factor determining the dynamics/kinetics of NPQ.

Recent findings evidenced that only separated LHCIIs and light-induced lumen acidification are the crucial factors that are necessary for qE (Johnson, 2020; Saccon et al., 2020). Therefore, it is natural to expect that in plants exposed to stress factors that lead to the detachment of LHCII from PSII supercomplexes, the dynamics of qE will be affected. Indeed, faster induction of qE has been observed for example in plants preheated in the dark (Ilík et al., 2010), where the disassembly of PSII supercomplexes is well known (e.g. Lípová et al., 2010). This scenario matches also the results obtained in this work for the *lhcb6* and *lhcb3 lhcb6* Arabidopsis mutants. As the C_2_S_2_M_2_ supercomplexes are replaced by C_2_S_2_ and a large pool of separated LHCII trimers, the dissociation is already achieved and therefore we can observe very fast qE induction in the first seconds of illumination, even though the overall rate of electron transport is retarded. These results are supported by a paper by Townsend et al. (2018), who have observed pronounced initial phase of qE induction in Arabidopsis mutants NoM that lack minor PSII antenna complexes and have a large pool of free LHCIIs. Taking into account the facts above, it appears that PSII arranged into C_2_S_2_ arrays with a pool of detached LHCII trimers at the array margins represents the arrangement that is “pre-prepared” for the formation of quenching centers and thus is beneficial for very fast induction of NPQ.

## Conclusions

Our experiments have shown that the loss of LHCB3 has surprisingly strong destabilizing effect on C_2_S_2_M_2_ supercomplexes, as the binding of the LHCB3-less M trimer to C_2_S_2_ is very weak. A very small part of the PSII supercomplexes in Arabidopsis *lhcb3* mutant appeared to adopt unique structure previously observed only in Norway spruce (“spruce-type” supercomplex), where LHCB6 is missing but the LHCB3-less M trimer is still attached to the PSII core. The absence of the “spruce-type” PSII supercomplexes in the *lhcb6* and *lhcb3 lhcb6* mutants indicates that on the contrary to spruce, in Arabidopsis both LHCB3 and LHCB6 proteins are needed for stable binding of the M trimer to PSII core. As the minor antenna LHCB4 is in direct contact with both the M trimer and LHCB6, we can speculate that the clue to the different stability of the “spruce-type” PSII supercomplex in Arabidopsis and spruce could be different isoform of this protein. The only isoform of LHCB4 in spruce is of LHCB4.3 type (renamed LHCB8), which is characteristic by the loss of a highly conserved motif at its C-terminus. On the other hand, in WT Arabidopsis as well as in all the analyzed mutants, the most populated isoforms were LHCB4.1 and LHCB4.2, with only negligible contribution of LHCB4.3. Further studies are needed to identify factors that are crucial for the formation and stabilization of PSII supercomplex with “spruce-like” structure in Arabidopsis. We are just beginning to understand the unique physiological benefits of the “spruce-like” PSII structure and more effort will be necessary to fully fathom the reasons that led a group of plants to “abandon” the widely conserved and evolutionary optimized PSII structure adopted by all other land plants.

PSII supercomplexes in Arabidopsis *lhcb6* and *lhcb3 lhcb6* mutants were present almost exclusively in the C_2_S_2_ form, which in WT plants appears primarily at high light conditions when LHCB3 and LHCB6 are downregulated. The C_2_S_2_ supercomplexes were arranged into very large semi-crystalline arrays, which can be connected with some interesting physiological features we have observed in *lhcb6* and *lhcb3 lhcb6* plants. These mutants showed fast activation of photosynthetic control of electron transport in thylakoid membranes, which can protect PSI against photoinhibition upon a sudden rise in light intensity, and even faster induction of NPQ, protecting PSII against overexcitation. Both these responses, which would be especially helpful in fluctuating light conditions, are probably associated with the restriction of electron transport between PSII and PSI resulting from the semi-crystalline arrangement of C_2_S_2_. However, on the contrary to the previous study on the *lhcb6* mutant (de Bianchi et al., 2008), we show that this restriction is only transient, as both PSI and PSII electron transport rates in *lhcb6* and *lhcb3 lhcb6* reach WT values after approximately 4 min of continuous illumination. It is tempting to hypothesize that this transient slowdown in electron transport between PSII and PSI could be controlled by light-dependent rearrangement of C_2_S_2_ semi-crystalline arrays, which would also explain the considerable variability in the types of C_2_S_2_ arrays we have observed in grana membranes of *lhcb3 lhcb6* mutants. Detailed structural analysis of the dynamics of C_2_S_2_ arrays in response to light could further contribute to the uncovering of the still enigmatic function of PSII crystalline arrangement in plants.

## Material and methods

### Plant material and growth conditions

Arabidopsis (*A.* *thaliana*) WT (accession Columbia) and T-DNA insertion lines *lhcb3* (SALK_020314c), *lhcb6* (SALK_077953) were obtained from Arabidopsis Biological Resource Center collection. Plants carrying double mutation (*lhcb3 lhcb6*) were prepared by crossing homozygous *lhcb3* and *lhcb6* plants. Plants from the F_1_ generation were self-fertilized and double homozygous plants were selected from the F_2_ generation via PCR genotyping (Thermo Scientific Phire Plant Direct PCR Kit) using the combination of gene-specific primers and universal T-DNA primer LBb1. Primer sequences were as follows: lhcb3_FP: AGAATTCCCTGGCGATTATGG; lhcb3_RP: ATAAAGGTCGTCACCGGAAATG; lhcb6_FP: GGTGAGGAACGAAGAACCAA; lhcb6_RP: CCAAACTCCCGACTTTACCA; and LBb1: GCGTGGACCGCTTGCTGCAACT. Arabidopsis plants were grown in a walk-in phytoscope (Photon Systems Instruments, Drásov, Czech Republic) at 22 °C, with an 8-h light/16-h dark cycle (light intensity 120 μmol photons m^−2^ s^−1^), and 60% humidity. All experiments were performed with 6–7-week-old plants. Barley (*H.* *vulgare*) mutant *viridis zb63* was grown hydroponically (Knop solution) in a growth chamber (PGC-40L2, Percival Scientific, USA) at 21 °C, with a 16-h light/8-h dark cycle (light intensity 100 μmol photons m^−2^ s^−1^) and 50% humidity for 2 weeks.

### Fresh weight determination and isolation of thylakoid membranes

Prior to isolation, plants were dark-adapted for 30 min. Arabidopsis rosettes or primary leaves of barley were cut and used for the determination of fresh weight. Subsequently, thylakoid membranes were isolated using the protocol described by Dau et al. (1995). All procedures were performed under green light on ice or at 4 °C. The chlorophyll content in the ﬁnal thylakoid membrane suspension was determined spectrophotometrically by a pigment extraction in 80% acetone (Lichtenthaler, 1987).

### Pigment analysis

Leaves were collected from dark-adapted plants (30 min), weighed, and frozen in liquid nitrogen. After homogenization in 80% acetone with a small amount of MgCO_3_ and centrifugation (3,600 *g*, 5 min, 4°C), the obtained supernatant was used for spectrophotometric (Unicam UV 500, Thermo Spectronics, UK) determination of chlorophyll and carotenoid content (Lichtenthaler, 1987). Quantification of xanthophyll content (violaxanthin, antheraxanthin, zeaxanthin) was performed by a reversed-phase high-performance liquid chromatography (HPLC) using Alliance e 2695 HPLC System (Waters, Milford, MA, USA) equipped with 2,998 Photodiode Array detectors. The separation was carried out using a gradient system (1.5 mL min^−1^ at 25 °C) on a LiChrospher 100 RP-18 (5 µm) LiChroCART 250-4 (Merck, Darmstadt, Germany) with acetonitrile:methanol:water (87:10:3; v/v) and methanol:*n*-hexane (80:20; v/v) as solvent systems. Quantification of the xanthophylls was based on the comparison of their absorbance (441 nm violaxanthin, 446 nm antheraxanthin, and 454 nm zeaxanthin) with corresponding standards purchased from DHI Lab Products (Hørsholm, Denmark).

### Western blot analysis

Thylakoid membranes (100 μg of chlorophyll) were mixed with 1 mL of extraction buffer (14 mM dl-dithiothreitol, 28 mM Na_2_CO_3_, 175 mM sucrose, 5% (w/v) SDS, and 10 mM EDTA-Na_2_), incubated at 70 °C for 30 min, and centrifuged for 10 min at 19,200 g. The supernatant containing isolated proteins was used for blotting. Isolated proteins (corresponding to 1 μg of chlorophyll) were supplemented with sample buffer (Tricine Sample Buffer, BioRad; 3× diluted) and dH_2_O to a total volume of 20 μL. After 10 min incubation at 70 °C, samples were loaded onto 10% gel (Mini-PROTEAN TGX Precast Protein Gel, Bio Rad, Hercules, USA). Electrophoretic buffers were prepared according to Schägger (2006). Electrophoresis was performed at RT with a constant voltage of 100 V for 45 min.

The separated proteins were transferred to a polyvinylidene fluoride membrane using Trans-Blot Turbo RTA Mini 0.2 µm PVDF Transfer Kit (Bio Rad) and detected using specific antibodies. All antibodies used in the present study were purchased from Agrisera (Vännäs, Sweden). The presence of primary antibodies Anti-LHCB3 (AS01 002) and Anti-LHCB6 (AS01 010) was detected with a secondary antibody with conjugated HRP enzyme and a chemiluminescent signal was recorded after developing with Immobilon Western Chemiluminescent HRP Substrate (Merck, Darmstadt, Germany) and visualized using gel scanner Amersham Imager 600RGB (GE HealthCare Life Sciences, Tokyo, Japan).

### CN-PAGE

CN-PAGE (CN-page) was performed according to Nosek et al. (2017) with minor modifications. Thylakoid membranes (10 μg of chlorophyll) were solubilized with *n*-dodecyl α-d-maltoside using a detergent:chlorophyll mass ratio of 15, and supplemented with sample buffer (50 mM HEPES/NaOH, pH 7.2, 0.4 M sucrose, 5 mM MgCl_2_, 15 mM NaCl, 10% glycerol) to a ﬁnal volume of 30 μL. After short gentle mixing (approximately for 2 s), samples were immediately centrifuged at 20,000 g/4 °C for 10 min to remove non-solubilized membranes. The supernatant was loaded onto a polyacrylamide gel with 4%–8% gradient (Wittig et al., 2007), stacking gel was not used. The electrophoretic separation was conducted in a Bio-Rad Mini protean tetra cell system (Bio Rad), starting with a constant current of 3.5 mA for 15 min and then continuing with a constant current of 7 mA until the front reached the bottom of the gel. The CN-PAGE gel was analyzed using a gel scanner Amersham Imager 600RGB (GE HealthCare Life Sciences, Tokyo, Japan). Transmission mode using white light illumination was used for the visualization of all bands.

### Mass spectrometry analysis

Isolated thylakoid membranes were subjected to filter-aided sample preparation as described elsewhere (Wiśniewski et al., 2009). The resulting peptides were analyzed by liquid chromatography–tandem mass spectrometry (LC–MS/MS) performed using UltiMate 3000 RSLCnano system (Thermo Fisher Scientific, Waltham, USA) on-line coupled with Orbitrap Elite hybrid spectrometer (Thermo Fisher Scientific).

Bands with desired PSII supercomplexes separated by CN-PAGE were excised and after washing procedures, each gel band was incubated with trypsin. LC–MS/MS analysis was performed using UltiMate 3000 RSLCnano system (Thermo Fisher Scientific) on-line coupled with Orbitrap Q Exactive HF-X spectrometer (Thermo Fisher Scientific). See the section Supplemental Methods S1 for full details regarding the analyses and data evaluation.

### EM and single particle analysis

Elution of isolated PSII supercomplexes from the gel and preparation of specimens for single particle EM was performed according to a procedure described by Kouřil et al. (2014). Electron micrographs were collected using a Tecnai G2 F20 microscope (FEI Technologies, Hillsboro, USA) with an Eagle 4K CCD camera (FEI Technologies) at 133,000× magnification. The pixel size at the specimen level after binning the images to 2,048 × 2,048 pixels was 0.218 nm. Approximately 92.000, 293.000, 42.000, 33.000, and 61.000 PSII projections were picked in semi-automated mode from 2,128, 7,642, 4,447, 2,311, and 1,925 micrographs of specimens prepared from the gel bands assigned as WT C_2_S_2_M_2_, WT C_2_S_2_M, *lhcb3* C_2_S_2_M, *lhcb6* C_2_S_2_M, and *lhcb3 lhcb6* C_2_S_2_, respectively. Individual datasets were subjected to reference-free two-dimensional classification using SCIPION image processing framework (de la Rosa-Trevín et al., 2016). The structure of the C_2_S_2_M_2_ supercomplex (van Bezouwen et al., 2017) was used to fit the projection maps of analyzed PSII supercomplexes.

EM of isolated grana membranes from *A.* *thaliana* WT, *lhcb3*, *lhcb6*, and *lhcb3 lhcb6* mutants, isolated according to Kouřil et al. (2013), was performed on a Jeol JEM2010 (Jeol, Tokyo, Japan) with a Quemesa CCD camera (EMSIS, Muenster, Germany) and a Jeol 2100 (Jeol, Japan) with a Tengra CCD camera (EMSIS, Muenster, Germany). Sub-areas (1,320 × 1,320 Å, 2,160 × 2,160 Å) of two-dimensional crystalline arrays of PSII C_2_S_2_ supercomplexes from *lhcb3 lhcb6* were analyzed using a single particle approach using RELION software (Scheres, 2012). The structure of the C_2_S_2_ supercomplex (Wei et al., 2016) was used to fit the two-dimensional arrays. Lattice parameters of the crystalline arrays and a ratio of the area of semi-crystalline PSII arrays per total area of the grana membranes were analyzed using ImageJ software (Schneider et al., 2012).

### Chlorophyll fluorescence and P700 measurements

Minimal chlorophyll fluorescence *F*_O_, maximal chlorophyll fluorescence *F*_M_, and maximum quantum yield of PSII photochemistry *F*_V_/*F*_M_ (where *F*_V_ = *F*_M_ – *F*_O_) for the dark-adapted state were evaluated from a fast chlorophyll fluorescence induction transients measured for 5 s on pre-darkened (30 min) Arabidopsis leaves using a Plant Efficiency Analyzer—PEA (Hansatech, King’s Lynn, Norfolk, UK). The parameters were calculated using Biolyzer software (R.M. Rodriguez, University of Geneva, Switzerland). Chlorophyll fluorescence was excited using red light adjusted to the relative intensity of 45%.

PSII and PSI functions were simultaneously recorded on whole leaves (pre-darkened for 30 min) using a Dual-PAM100 measuring system (Heinz Walz, Effeltrich, Germany) during light exposure by red actinic light (800 μmol photons m^−2^ s^−1^) for 16 min and using 300 ms saturating red light pulses (10,000 μmol photons m^−2^ s^−1^). PSII function and induction of NPQ in PSII were detected and calculated as the effective yield of PSII photochemistry for light-adapted state Y(II) = (*F*_M_′ – *F*)/*F*_M_′ (*F*_M_′ is the maximum chlorophyll fluorescence intensity at a light-adapted state and *F* is related chlorophyll fluorescence level at the state induced by the actinic light) and NPQ = (*F*_M_ – *F*_M_′)/*F*_M_′ (*F*_M_ is the maximum chlorophyll fluorescence intensity at dark-adapted state; Genty et al., 1989; Bilger and Björkman, 1990). Parameters related to PSI function, Y(I), Y(ND), and Y(NA), that is the effective quantum yield of PSI photochemistry, and the quantum yields of non-photochemical energy dissipation due to donor and acceptor side limitation, respectively, were calculated using the Dual-PAM100 software according to Klughammer and Schreiber (2008). The electron transport rate through PSII and PSI (ETR-II and ETR-I) are directly related to Y(II) a Y(I), respectively (= PAR × Y(II) or Y(I) × 0.84 × 0.5, where PAR is the irradiation level at 400–700 nm and the constants represent the assumed average leaf absorptance of PAR and the fraction of the light absorbed by given photosystem). The fraction of reduced PQ pool in thylakoid membranes was estimated as 1−qP, where qP is the photochemical quenching coefficient calculated as (*F*_M_′−*F*)/(*F*_M_′−*F*_O_′). *F*_O_′ represents related minimal chlorophyll fluorescence level during the light exposure and was calculated according to Oxborough and Baker (1997).

### Estimation of effective antenna size of PSII

Chlorophyll fluorescence induction was measured with a Dual-PAM100 system (Heinz Walz) on leaves that were excised from dark-adapted (30 min) Arabidopsis plants and subsequently infiltrated with 50 µM 3-(3,4-dichlorophenyl)-1,1-dimethylurea (DCMU) using five low-pressure shockwaves. This treatment was sufficient to block the PSII electron transport from *Q*_A_ to *Q*_B_. The fluorescence induction was excited by red light (12 µmol photons m^−2^s^−1^) according to Belgio et al. (2014). Evaluation of effective absorption cross-section (*σ*, denoted as ACS PSII in Table 3) of PS II in the samples was estimated from fluorescence induction curves measured for 450 ms and double-normalized to obtain relative variable Chl fluorescence *V*(*t*) as

$$V\left(t\right)=\left(F\left(t\right)–F_{0}\right)/\left(F_{M}–F_{0}\right),$$

where *F*_0_ and *F*_M_ are minimal and maximal Chl fluorescence, respectively, and *F*(*t*) is Chl fluorescence at time *t*.

According to the theory of Malkin et al. (1981), the complementary area (CA, area between the *V*(*t*) measured with DCMU-treated samples and the horizontal line at the *F*_M_ level) of dark-adapted sample is related to *σ* as follows:

CA=1/σI,

where *I* is the incident light intensity. Since *I* was the same for all measured samples, we get

σ≈1/CA.

The CA has been calculated using Microsoft Excel.

Another estimation of effective antenna size of PSII of the samples was performed from the measurement of chlorophyll fluorescence curves of dark-adapted (30 min) leaves (without DCMU) measured using a PEA (Hansatech) under high intensity of incident light (adjusted to 45%). Under these conditions, a typical O–J–I–P Chl fluorescence induction curve is measured (Strasser and Srivastava, 1995; Lazár, 2006). According to the theory of energy fluxes and the JIP test (Strasser et al., 2004; Stirbet et al., 2018), maximal trapping flux at time zero TR_0_/RC (corresponding to the rate by which an incident light is trapped by the reaction centers of PSII resulting in the reduction of *Q*_A_) can be expressed as

TR_0_/RC = *M*_0_/*V*_J_,where *M*_0_ and *V*_J_ are defined as follows:

$$M_{0}=4\left(F_{300µs}–F_{50µs}\right)/\left(F_{M}–F_{0}\right)$$

$$V_{J}=\left(F_{J}–F_{0}\right)/\left(F_{M}–F_{0}\right),$$

where *F*_50µs_, *F*_300µs_, and *F*_J_ are values of Chl fluorescence signal at 50 and 300 µs and at the position of the J step (at 2 ms), respectively, of the O–J–I–P curve. Since the initial rate of *Q*_A_ reduction reflects the effective antenna size of PSII of the sample (Lazár et al., 2001), TR_0_/RC was used as another way to estimate the effective antenna size of PSII. The TR_0_/RC was calculated using Biolyzer software (R.M. Rodriguez, University of Geneva, Switzerland).

### Measurement of state transitions

State transitions were induced and monitored via the measurement of chlorophyll fluorescence using a Dual-PAM100 (Heinz Walz) according to de Bianchi et al. (2008) with modifications. A measurement protocol started with a preferential excitation of PSII by illumination with red light (13 μmol photons m^−2^ s^−1^) for 15 min. Then PSI was excited by far-red light for 15 min simultaneously with the red light, which was followed by 800 ms saturating red light pulse (10,000 μmol photons m^−2^ s^−1^) to determine the *F*_M_′ level in State I. A transition from State I to State II was achieved by red light illumination for 15 min, followed by a saturating light pulse to determine the *F*_M_″ level in State II. The half-time of state transition from State I to State II was evaluated as the half-time of a gradual fluorescence decay upon switching off far-red light according to Damkjær et al. (2009). Parameter qT, which reflects the decrease in the LHCII antenna size, was calculated as (*F*_M_′−*F*_M_″)/*F*_M_′ according to Ruban and Johnson (2009).

### CD spectroscopy

Room temperature CD spectra of intact leaves were recorded in the range of 400–750 nm with a J-815 spectropolarimeter (Jasco, Tokyo, Japan). Intact leaves were supported by a flat cell and CD spectra were measured perpendicularly to the optical path. Measurements were carried out at room temperature with 0.5 nm step, 1 s integration time, 3 nm band-pass, and scanning speed 100 nm min^−1^. To improve the signal-to-noise ratio, leaves were infiltrated with distilled water prior to the measurements using a 2-min interval at low pressure, and three scans were collected and averaged. CD spectra were normalized to the Chl *Q*_y_ absorption band. In order to minimize the influences of the overlapping excitonic CD bands, the amplitudes of the (+)685 nm and (+)505 nm psi-type CD bands were calculated as the difference between the CD signal at 685 and 750 nm and between 505 and 620 nm, respectively.

### Chlorophyll fluorescence decay after a single-turnover saturating flash

The kinetics of the Chl fluorescence decay after a single-turnover saturating flash was monitored using Joliot-type kinetic spectrometer JTS-100 (Biologic, Seyssinet-Pariset, France). Arabidopsis plants were dark-adapted for 30 min, individual leaves were detached and immediately used for the measurement. Single turnover saturating (0.5 J) actinic flashes of 2 μs duration at half-peak intensity were provided by a xenon lamp (Hamamatsu LF1 L-11730-04-01-1, Shimokanzo, Japan) with Schott BG39 filter (Schott, Mainz, Germany), whereas the instruments LED system with a narrow bandpass filter centered at 650 nm (XHQA650; FWHM of 12 nm) provided measuring flashes. Fluorescence decay was recorded in the time range 15 μs to 50 s. Multicomponent deconvolution of the obtained fluorescence decay curves was achieved by fitting the experimental data with two exponential components (fast and middle phase) and one hyperbolic component (slow phase) as described earlier (Vass et al., 1999):

$$F_{V}=A_{0}+A_{1}\cdot e^{\frac{-t}{T_{1}}}+A_{2}\cdot e^{\frac{-t}{T_{2}}}+\frac{A_{3}}{1+\frac{t}{T_{3}}}$$

where *F*_V_ = *F*(*t*) – *F*_0_, *F*(*t*) is the fluorescence yield at time *t*, *F*_0_ is the basic fluorescence level before the flash, *A*_1_–*A*_3_ are the amplitudes, *T*_1_–*T*_3_ are the time constants and *A*_0_ describe non-decaying fluorescence component in the time span of the measurement.

## Accession numbers

The accession numbers are as follows: LHCB3 (AT5G54270), LHCB6 (AT1G15820), LHCB4.1 (AT5G01530), LHCB4.2 (AT3G08940), and LHCB4.3 (AT2G40100). The mass spectrometry proteomic data have been deposited to the ProteomeXchange Consortium via the PRIDE partner repository with the identifiers: PXD023071 (thylakoid membranes) and PXD026019 (PSII supercomplexes).

## Supplemental data

**Supplemental Figure S1.** Relative content of light-harvesting proteins in PSII supercomplexes separated by CN-PAGE.

**Supplemental Figure S2.** Gallery of electron micrographs of grana membranes isolated from Arabidopsis WT, *lhcb3* and *lhcb6* mutants.

**Supplemental Figure S3.** Electron micrograph of grana membranes isolated from Arabidopsis *lhcb3 lhcb6* mutant.

**Supplemental Figure S4.** Electron micrograph of grana membranes isolated from Arabidopsis *lhcb3 lhcb6* mutant—regular arrays.

**Supplemental Figure S5.** Electron micrograph of grana membranes isolated from Arabidopsis *lhcb3 lhcb6* mutant—carpet-like motive.

**Supplemental Figure S6.** Kinetics of *Q*_A_^−^ reoxidation following a single turnover saturating flash.

**Supplemental Figure S7.** Measurements of state transitions in WT and mutant plants (*lhcb3*, *lhcb6*, *lhcb3 lhcb6*).

**Supplemental Table S1.** Decay kinetics of flash-induced variable fluorescence in Arabidopsis leaves.

**Supplemental Methods S1.** Mass spectrometry analysis of isolated thylakoid membranes and PSII supercomplexes.

## Supplementary Material

kiab396_Supplementary_Data

## Notes

R.K., I.I., and P.I. planned and designed the research. All authors performed experiments and analyzed the data. I.I., R.K., and P.I. wrote the manuscript with input from all authors, and all authors revised and approved it.

The author responsible for distribution of materials integral to the findings presented in this article in accordance with the policy described in the Instructions for Authors (https://academic.oup.com/plphys/pages/General-Instructions) is: Iva Ilíková (ilikova@ueb.cas.cz).
