# Corrigendum to: Towards spruce-type photosystem II: consequences of the loss of light-harvesting proteins LHCB3 and LHCB6 in Arabidopsis

**DOI:** 10.1093/plphys/kiab505

**Published:** 2021-11-11

**Authors:** Iva Ilíková, Petr Ilík, Monika Opatíková, Rameez Arshad, Lukáš Nosek, Václav Karlický, Zuzana Kučerová, Pavel Roudnický, Pavel Pospíšil, Dušan Lazár, Jan Bartoš, Roman Kouřil

*Plant Physiology*, https://doi.org/10.1093/plphys/kiab396

In the originally published version of this manuscript, the affiliations of Pavel Pospíšil and Dušan Lazár were incorrect.

The affiliation of both Pavel Pospíšil and Dušan Lazár is:

“Department of Biophysics, Centre of the Region Haná for Biotechnological and Agricultural Research, Palacký University, 783 71 Olomouc, Czech Republic.”

This error has been corrected.

