# Supplementary material for: Towards spruce-type photosystem II: consequences of the loss of light-harvesting proteins LHCB3 and LHCB6 in Arabidopsis
Source: Plant Physiol. 2021 Sep 1;187(4):2691–715. doi: 10.1093/plphys/kiab396 (PMC8644234; doi:10.1093/plphys/kiab396)
Supplement: kiab396_Supplementary_Data [file kiab396_supplementary_data.pdf]

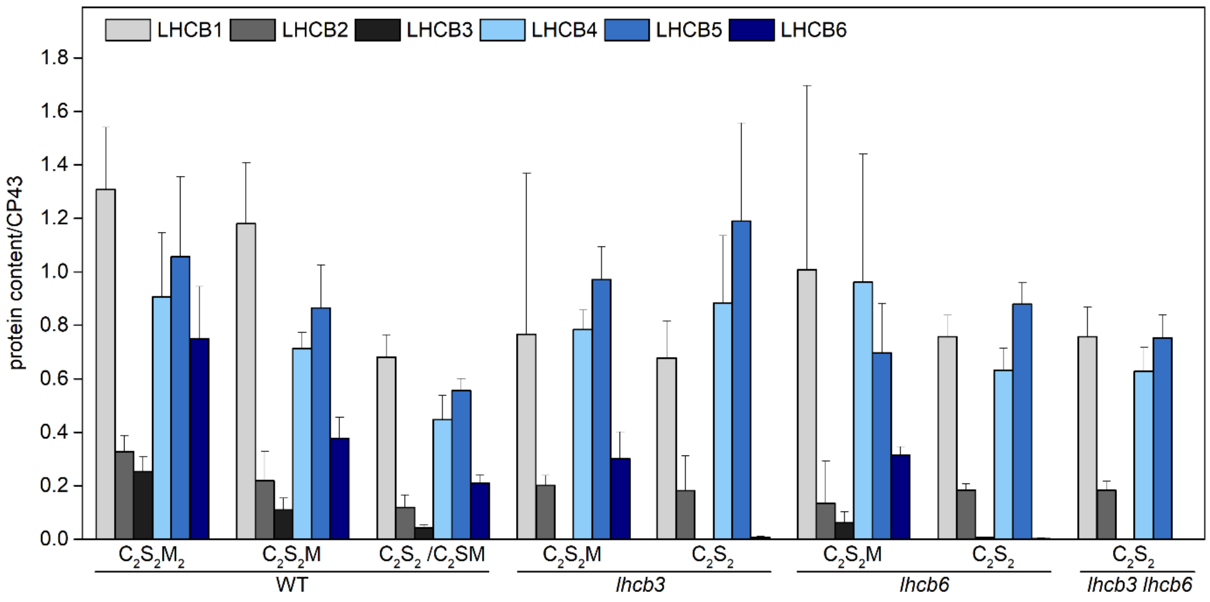

Supplemental Figure S1. Relative Content of Light-harvesting Proteins in PSII supercomplexes separated by CN-PAGE

The content of individual light-harvesting proteins Lhcb1-6 was determined in samples prepared via the elution of excised gel bands separated from wild type (WT) and mutant plant (*lhcb3, lhcb6, lhcb3 lhcb6*) thylakoids via CN-PAGE (see Figure 3A). The content of individual proteins was analyzed using liquid chromatography-tandem mass spectrometry (LC-MS/MS) and evaluated based on relative PG intensities relatively to the content of chlorophyll protein 43 (CP43, the inner antenna of PSII). The presented values are means ± SD from 4 replicates. These data complement Figure 11 from the main text. Different forms of separated PSII supercomplexes consist of PSII core dimer (C_2) and one and/or two copies of strongly (S) and moderately (M) bound light-harvesting trimers.

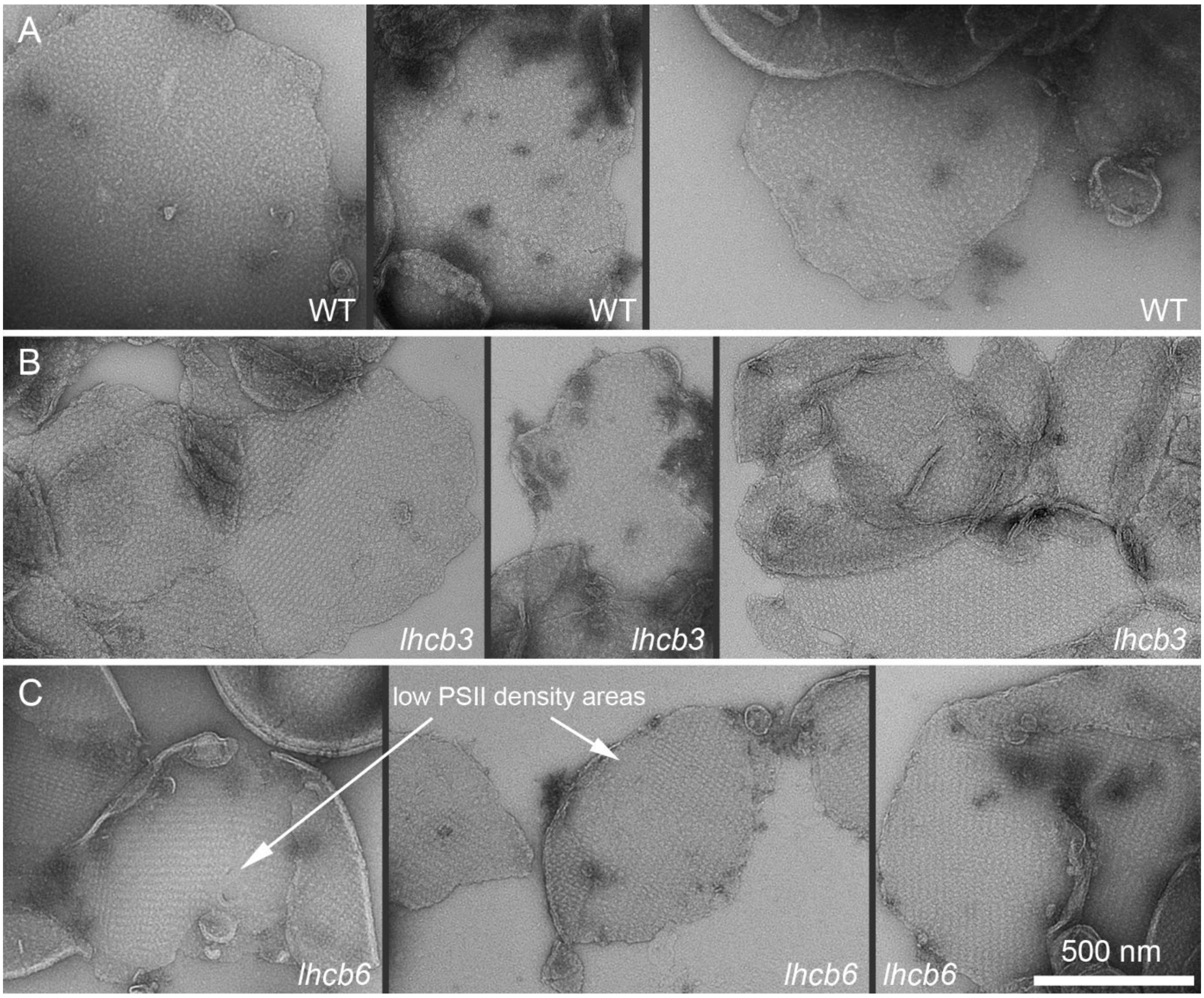

Supplemental Figure S2. Gallery of Electron Micrographs of Grana Membranes Isolated from Arabidopsis WT, *lhcb3* and *lhcb6* Mutants

(A, B) Organization of PSII complexes in the grana membranes from Arabidopsis WT and *lhcb3* mutant is predominantly random. Occasionally, PSII complexes are organized into semi-crystalline arrays.

(C) PSII complexes in the grana membranes from Arabidopsis *lhcb6* mutant are organized into semi-crystalline arrays. In addition, there are often areas at the periphery of the arrays, where PSII complexes are less ordered and their density is lower, likely due to a presence of free LHCII. This gallery supports Figure 7 from the main text.

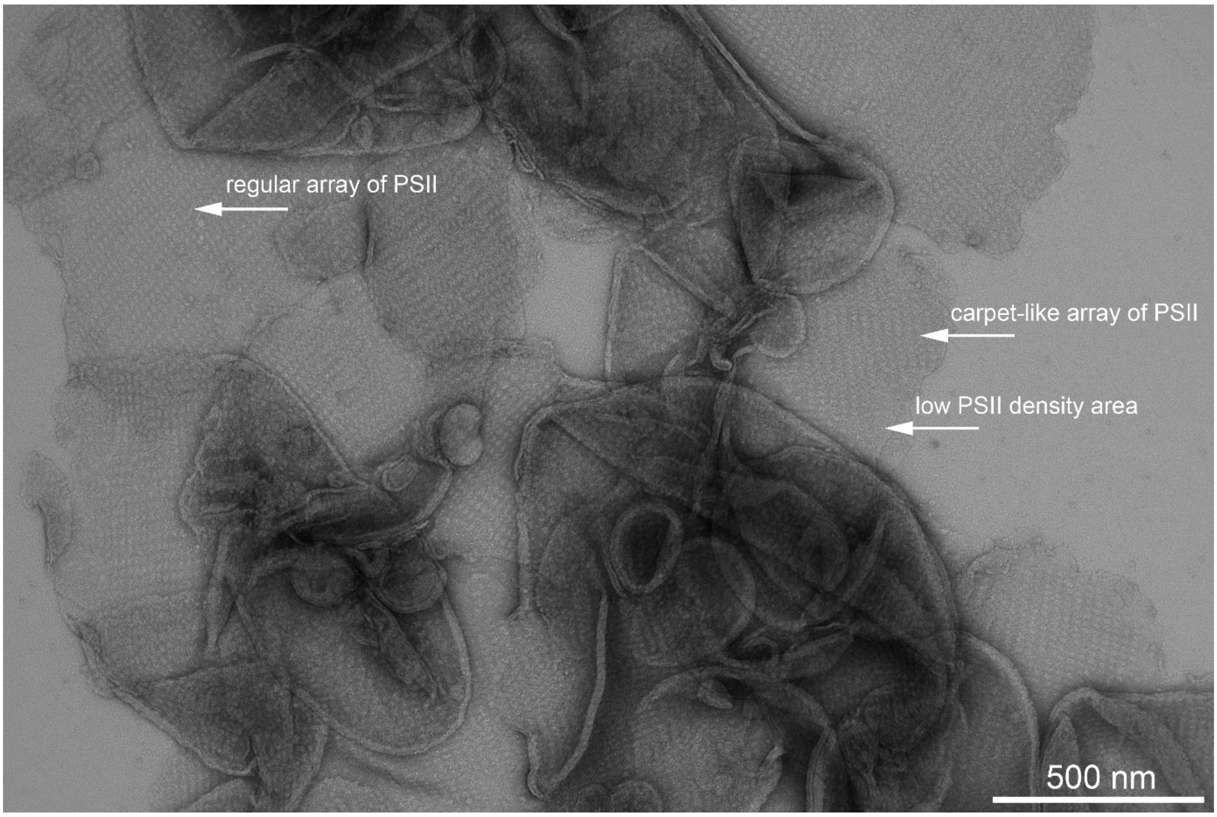

Supplemental Figure S3. Electron Micrograph of Grana Membranes Isolated from Arabidopsis *lhcb3 lhcb6* Mutant

The image shows that PSII complexes are almost exclusively organized into large semi-crystalline arrays. Two types of arrays can be distinguished - regular and carpet-like arrays. Rarely, there are regions in the membrane, where PSII complexes are less ordered and their density is lower, likely due to a presence of free LHCII. This figure complements Figure 5 from the main text.

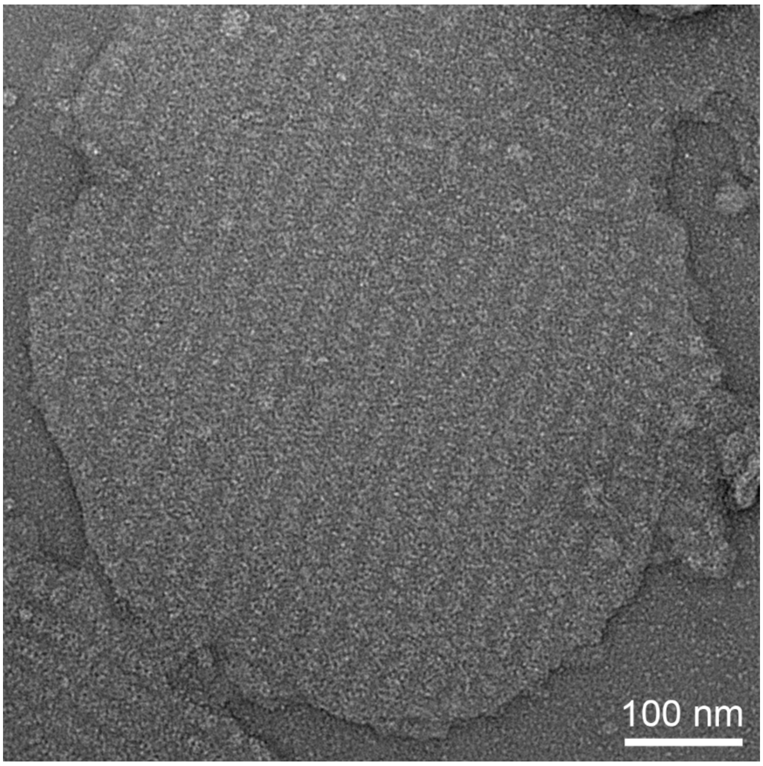

Supplemental Figure S4. Electron Micrograph of Grana Membranes Isolated from Arabidopsis *lhcb3 lhcb6* Mutant – Regular Arrays

A raw electron micrograph, which was used for image analysis of selected sub-areas (1320x1320 Å) of ordered arrays of PSII complexes. The result of image analysis, which revealed the regular array of C_2S_2 supercomplexes, is presented in the Figure 5A, B.

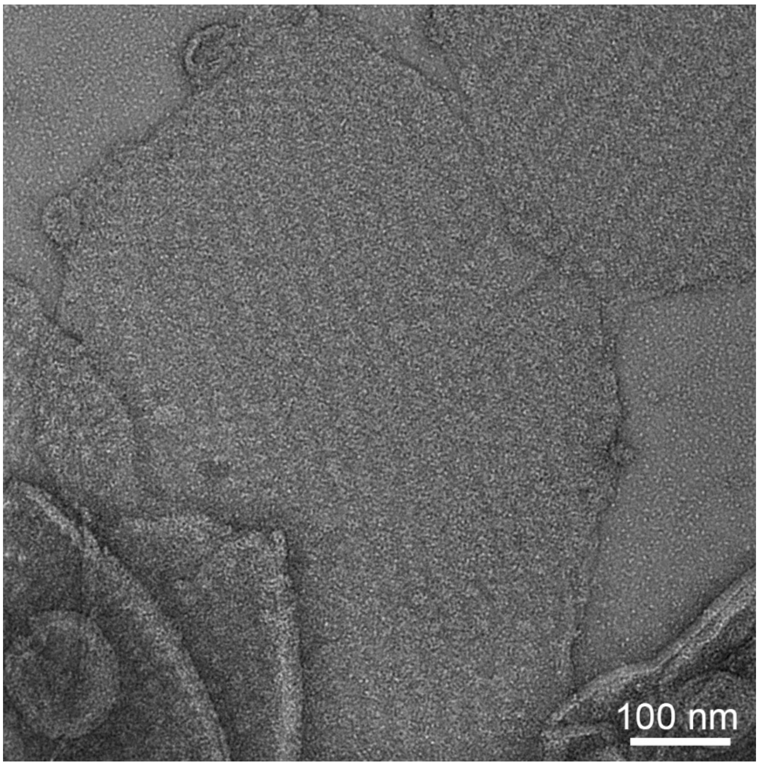

Supplemental Figure S5. Electron Micrograph of Grana Membranes Isolated from Arabidopsis *lhcb3 lhcb6* Mutant – Carpet-Like Motive

A raw electron micrograph, which was used for image analysis of selected sub-areas (2160x2160 Å and 1230x1230 Å) of ordered arrays of PSII complexes. Results of image analysis, which revealed the array of C_2S_2 supercomplexes with carpet-like motive, are presented in the Figure 5C-F.

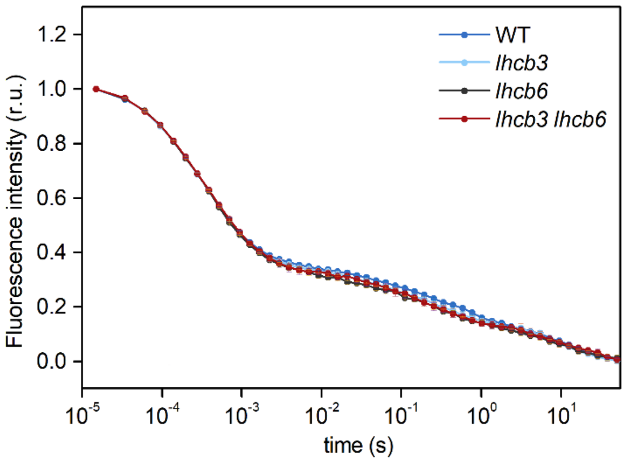

Supplemental Figure S6. Kinetics of Q_A^- Reoxidation Following a Single Turnover Saturating Flash

Fluorescence decay kinetics were measured in dark-adapted leaves (30 min) in wild type (WT) and *lhcb3*, *lhcb6* and *lhcb3 lhcb6* mutants. In order to facilitate a comparison of the kinetics, the fluorescence signal was subtracted by F_0 level detected before the measurement and then normalized to the maximum fluorescence level at 15μs (the first detected fluorescence value after the single turnover flash). This fluorescence decay reflects Q_A^- reoxidation in PSII after a single turnover saturating flash. Averaged curves for given sample type are presented including ± SD and calculated from 4-6 replicates. The measured kinetics were deconvoluted by three decay components (see Supplemental Table S1). The data complement 6th paragraph of Results.

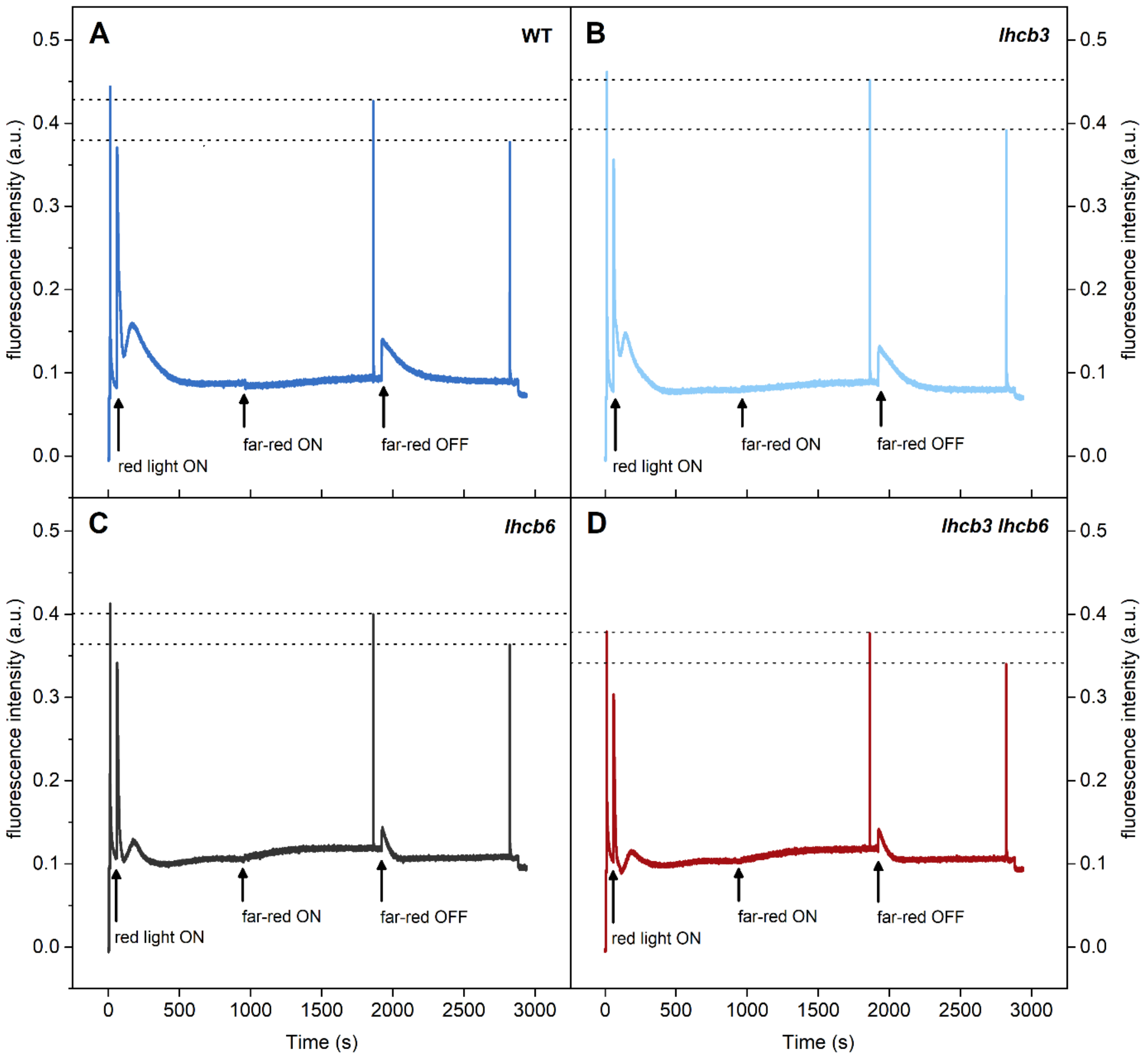

Supplemental Figure S7. Measurements of State Transitions in Wild Type (WT) and Mutant Plants (*lhcb3, lhcb6, lhcb3 lhcb6*)

State transitions were induced in dark-adapted leaves of Arabidopsis WT **(A)**, *lhcb3* **(B)**, *lhcb6* **(C)**, and *lhcb3 lhcb6* **(D)** by illumination with red light (13 µmol photons m^{-2} s^{-1}). State I was induced by additional illumination with far-red light, transition to State II was induced by switching off the far-red light. Maximum fluorescence F_M in the dark-adapted state, $F_{M'}$ in State I, and $F_{M''}$ in State II were induced by saturation light pulses (10.000 µmol photons m^{-2} s^{-1}, 800 ms). The data supplement Table 4 from the main text.

Supplemental Table S1. Decay Kinetics of Flash-Induced Variable Fluorescence in Arabidopsis Leaves

Multicomponent deconvolution of the fluorescence decay curves presented in Supplemental Figure S6. The kinetics was fitted by two exponential components (fast and middle phase) and one hyperbolic component (slow phase). For each component, T is the characteristic time constant and A is the corresponding amplitude. Presented values are means ± SD (*n* = 4–6). The data complement 6th paragraph of Results.

	Fast phase T_1(μs)/A_1(%)	Middle phase T_2(ms)/A_2(%)	Slow phase T_3(s)/A_3(%)
WT	459 ± 25/62.7 ± 0.5	80 ± 11/14.7 ± 0.4	3.63 ± 0.18/22.6 ± 0.4
lhcb3	459 ± 7/63.1 ± 0.5	70 ± 5/15.6 ± 1.1	3.48 ± 0.42/21.2 ± 1.1
lhcb6	477 ± 21/64.4 ± 0.7	69 ± 8/15.8 ± 0.7	3.92 ± 0.79/19.8 ± 1.4
lhcb3 lhcb6	486 ± 10/63.8 ± 1.4	81 ± 5/16.2 ± 0.7	4.04 ± 0.37/20.0 ± 1.6

Supplemental Methods S1: Mass spectrometry analysis of isolated thylakoid membranes and PSII supercomplexes

Thylakoid membranes were lysed in SDT buffer (4% SDS, 0.1M DTT, 0.1M Tris/HCl, pH 7.6) in a thermomixer (Eppendorf ThermoMixer® C, 30 min, 95°C, 750 rpm). The sample was centrifuged (15 min, 20,000 x g) and the supernatant used for filter-aided sample preparation as described elsewhere (Wisniewski et al., 2009) using 1 µg of trypsin (sequencing grade; Promega). Resulting peptides were analysed by LC-MS/MS. LC-MS/MS analyses of all peptides were done using UltiMate 3000 RSLCnano system (Thermo Fisher Scientific) connected to Orbitrap Elite hybrid spectrometer (Thermo Fisher Scientific). Prior to LC separation, tryptic digests were online concentrated and desalted using trapping column (Acclaim™ PepMap™ 100 C18, dimensions 300 µm × 5 mm, 5 µm particles; Thermo Fisher Scientific, part number 160454). After washing of the trapping column with 0.1% formic acid, the peptides were eluted in backflush mode (flow 300 $nl.min^{-1}$) from the trapping column onto an analytical column (Acclaim™ PepMap™ 100 C18, 3 µm particles, 75 µm × 500 mm; Thermo Fisher Scientific, part number 164570) by 100 min gradient program (3-80% of mobile phase B; mobile phase A: 0.1% formic acid in water; mobile phase B: 0.1% formic acid in 80% acetone). The equilibration of the trapping and analytical column was done prior to sample injection to the sample loop. The analytical column outlet was directly connected to the Digital PicoView 550 (New Objective) ion source with sheath gas option and SilicaTip emitter (New Objective; FS360-20-15-N-20-C12). ABIRD (Active Background Ion Reduction Device, ESI Source Solutions) was installed. MS data were acquired in a data-dependent strategy selecting up to top 10 precursors based on precursor abundance in the survey scan (*m/z* 350–2,000). The resolution of the survey scan was 60,000 (at *m/z* 400) with a target value of $1×10^6$ ions and maximum injection time of 1,000 ms. HCD MS/MS spectra were acquired with a target value of 50,000 and resolution of 15,000 (at *m/z* 400). The maximum injection time for MS/MS was 500 ms. Dynamic exclusion was enabled for 45 s after one MS/MS spectra acquisition. The isolation window for MS/MS fragmentation was set to 2.0 *m/z*.

Photosystem II supercomplexes were manually excised from CN-PAGE gels and after washing procedures, each band was subjected to protein reduction (10mM DTT in 25mM $NaHCO_3$, 45 min, 56°C, 750 rpm) and alkylation (55mM IAA in 25mM $NaHCO_3$; 30 min, RT, 750 rpm) step. After further washing by 50% acetone/$NaHCO_3$ and pure acetone, the gel pieces were incubated with 125 ng trypsin (sequencing grade;

Promega) in 50mM $NaHCO_3$. The digestion was performed for 2 h at 40 °C in a thermomixer (Eppendorf ThermoMixer® C, 750 rpm). Tryptic peptides were extracted into LC-MS vials by 2.5% formic acid in 50% acetone with addition of polyethylene glycol (20,000; final concentration 0.001%) (Stejskal et al., 2013) and concentrated in a SpeedVac concentrator (Thermo Fisher Scientific). LC-MS/MS analyses of all peptides were done using RSLCnano system (UltiMate™ 3000, Thermo Fisher Scientific) connected to Orbitrap Q Exactive HF-X spectrometer (Thermo Fisher Scientific) as described above. MS data were acquired in a data-dependent strategy selecting up to top 15 precursors based on precursor abundance in the survey scan (*m/z* 350–2,000). The resolution of the survey scan was 120,000 (at *m/z* 200) with a target value of 3×10^6 ions and maximum injection time of 250 ms. MS/MS spectra were acquired with a target value of 50,000 and resolution of 15,000 (at *m/z* 200). The maximum injection time for MS/MS was 250 ms. Dynamic exclusion was enabled for 30 s after one MS/MS spectra acquisition. The isolation window for MS/MS fragmentation was set to 1.2 *m/z*.

For data evaluation, we used MaxQuant software (v1.6.17) (Cox and Mann 2008) with inbuild Andromeda search engine (Cox et al., 2011). Searches for samples of thylakoid membranes and photosystem II supercomplexes were done separately against protein databases of *Arabidopsis thaliana* (27,500 protein sequences, version from 12-08-2020, downloaded from ftp://ftp.uniprot.org/pub/databases/uniprot/current_release/knowledgebase/reference_proteomes/Eukaryota/UP000006548_3702.fasta.gz) and cRAP contaminants (112 sequences, version from 22-11-2018, downloaded from http://www.thegpm.org/crap). Modifications for all database searches were set as follows: oxidation (M), deamidation (N, Q), and acetylation (Protein N-term) as variable modifications, with carbamidomethylation (C) as a fixed modification. Enzyme specificity was tryptic with two permissible miscleavages. Only peptides and proteins with false discovery rate threshold under 0.01 were considered. Relative protein abundance was assessed using protein intensities calculated by MaxQuant. Intensities of reported proteins were further evaluated using software container environment (https://github.com/OmicsWorkflows/KNIME_docker_vnc; version 4.1.3a). Processing workflow is available upon request: it covers reverse hits and contaminant protein groups (cRAP) removal, protein group intensities log2 transformation and normalization (median). For the purpose of this article, protein groups reported by

MaxQuant are referred to as proteins. Mass spectrometry proteomics data were deposited to the ProteomeXchange Consortium via PRIDE (Perez-Riverol et al 2019) partner repository under dataset identifiers: PXD023071 (Thylakoid membranes) and PXD026019 (PSII Supercomplexes).

Supplemental References

Cox, J. and Mann, M. (2008). MaxQuant enables high peptide identification rates, individualized p.p.b.-range mass accuracies and proteome-wide protein quantification. Nat Biotechnol. **26**: 1367–1372.

Cox, J., Neuhauser, N., Michalski, A., Scheltema, R.A., Olsen, J.V. and Mann, M. (2001). Andromeda: A Peptide Search Engine Integrated into the MaxQuant Environment. J. Proteome Res. **10**: 1794–1805.

Perez-Riverol, Y., Csordas, A., Bai, J., Bernal-Llinares, M., Hewapathirana, S., Kundu, D.J., et al. (2019). The PRIDE database and related tools and resources in 2019: improving support for quantification data. Nucleic Acids Res. **47**: D442–D450.

Stejskal, K., Potěšil, D. and Zdráhal, Z. (2013). Suppression of Peptide sample losses in autosampler vials. J. Proteome Res. **12**: 3057–3062.

Wiśniewski, J.R., Zougman, A., Nagaraj, N. and Mann, M. (2009). Universal sample preparation method for proteome analysis. Nat. Methods **6**: 359–362.
